# The mTOR-S6 kinase pathway promotes stress granule assembly

**DOI:** 10.1038/s41418-018-0076-9

**Published:** 2018-03-09

**Authors:** Aristeidis P. Sfakianos, Laura E. Mellor, Yoke Fei Pang, Paraskevi Kritsiligkou, Hope Needs, Hussein Abou-Hamdan, Laurent Désaubry, Gino B. Poulin, Mark P. Ashe, Alan J. Whitmarsh

**Affiliations:** 1 School of Biological Sciences, Faculty of Biology, Medicine and Health, University of Manchester, Manchester Academic Health Science Centre, Michael Smith Building, Oxford Road, Manchester, M13 9PT UK; 2 Laboratory of Therapeutic Innovation (UMR 7200), CNRS, University of Strasbourg, 67401 Illkirch, France

**Keywords:** Cell biology, Genetics

## Abstract

Stress granules are cytoplasmic mRNA-protein complexes that form upon the inhibition of translation initiation and promote cell survival in response to environmental insults. However, they are often associated with pathologies, including neurodegeneration and cancer, and changes in their dynamics are implicated in ageing. Here we show that the mTOR effector kinases S6 kinase 1 (S6K1) and S6 kinase 2 (S6K2) localise to stress granules in human cells and are required for their assembly and maintenance after mild oxidative stress. The roles of S6K1 and S6K2 are distinct, with S6K1 having a more significant role in the formation of stress granules via the regulation of eIF2α phosphorylation, while S6K2 is important for their persistence. In *C. elegans*, the S6 kinase orthologue RSKS-1 promotes the assembly of stress granules and its loss of function sensitises the nematodes to stress-induced death. This study identifies S6 kinases as regulators of stress granule dynamics and provides a novel link between mTOR signalling, translation inhibition and survival.

## Introduction

Cells employ a number of mechanisms to adapt and survive stressful conditions [1, 2]. Many stresses cause repression of translation coincident with the formation of stress granules (SGs) [3, 4]. This is primarily mediated by the phosphorylation of translation initiation factor 2 alpha (eIF2α) by stress-responsive protein kinases [3–5]. Independent of this, SGs are induced by the inhibition of other translation factors, including the mRNA helicase eIF4A [6, 7]. SGs are large non-membrane bound cytoplasmic entities that contain mRNAs, translation initiation complexes and other RNA-binding proteins (RNPs) [3, 8]. Stress-induced translation inhibition leads to specific RNPs binding to stalled mRNAs in translation complexes and nucleating SGs by promoting transient interactions between their disordered regions [8–12]. Recent studies have indicated that SGs exist as liquid droplets with a solid core surrounded by a fluid shell and that they form via liquid–liquid phase separation dependent upon the local concentration of mRNAs and RNPs [13–17]. SGs are highly dynamic, triaging and storing mRNAs to enable cells to re-programme translation and mount a protective response [3, 8, 18]. In addition, there is accumulating evidence that SGs regulate cellular signalling pathways to coordinate changes in translation with altered cell fate [4, 19, 20]. Recent studies have indicated that the interplay between the mechanistic target of rapamycin (mTOR) pathway and SGs is important for regulating cell fate [21–23].

The mTOR protein kinase is a component of the mTORC1 complex that targets many proteins involved in translation [24]. It phosphorylates and inactivates 4EBP proteins causing them to release eIF4E for binding to eIF4G1 and thus permitting translation initiation [24]. It also phosphorylates the p70 S6 kinases S6K1 and S6K2, leading to their activation and phosphorylation of substrates that promote translation initiation, including S6 ribosomal protein (RPS6) [24]. In yeast, TORC1 activity is suppressed by its sequestration into SGs following heat stress and this protects against DNA damage [21]. Similarly, mTORC1 components are sequestered into mammalian SGs, resulting in reduced mTORC1 assembly and suppression of mTORC1 hyperactivation-induced apoptosis [22, 23]. Whilst the actions of mTORC1 can be inhibited by SGs, paradoxically, there is evidence that mTORC1 can suppress translation and facilitate SG assembly [25, 26]. Thus, it is important to gain a better understanding of the complex role of the mTOR pathway in SG dynamics. In this study, we demonstrate that the mTORC1 effector kinases S6K1 and S6K2 have distinct roles in SG assembly and maintenance in cultured human cells and that the S6 kinase orthologue, RSKS-1, promotes SG assembly in *Caenorhabditis elegans*.

## Results

### S6K1 and S6K2 localise to SGs

**Fig. 1:**
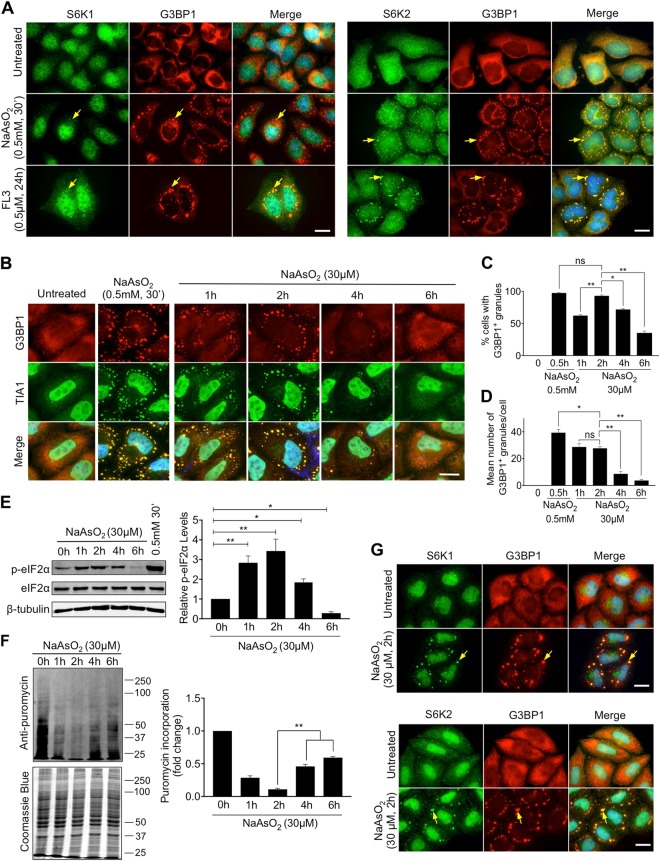
S6 kinases accumulate in stress granules. **a** HeLa cells were treated with either 0.5 mM of NaAsO_2_ for 30 min or 0.5 μM FL3 for 24 h and stress granules labelled using an antibody against G3BP1 (red). The localisation of S6K1 and S6K2 were observed by immunofluorescence staining (green). Nuclei were stained with DAPI (blue). Merged images are shown. Yellow arrows indicate examples of stress granules. Scale bar = 25 μm. **b** HeLa cells were treated with either 0.5 mM of NaAsO_2_ for 30 min or 30 μM NaAsO_2_ for 1, 2, 4 or 6 h. Stress granule formation was assessed by immunofluorescence staining of G3BP1 (red) or TIA1 (green). **c**, **d** Quantification of the % of cells containing stress granules after treatment and the mean number of stress granules in the cells displaying stress granules. 100 cells were counted in each of the 3 biological repeats. Scale bar = 20 μm. **e** HeLa cells were treated with either 0.5 mM of NaAsO_2_ for 30 min or 30 μM NaAsO_2_ for 1, 2, 4 or 6 h and protein extracts immunoblotted for phosphorylated eIF2α (p-eIF2α), eIF2α and β-tubulin. Quantification was performed from three independent experiments. **f** HeLa cells were treated with 30 μM NaAsO_2_ for 1, 2, 4 or 6 h. In the last 5 min of treatment, cells were incubated with 5 μg/ml puromycin. Incorporation of puromycin into newly synthesised protein was assessed by immunoblotting. Band intensities for each lane were measured in the biological repeats and normalised against intensity of Coomassie Blue staining. For **c**–**f**, error bars are s.e.m and the data were analysed using one-way Anova (*p < 0.04; **p < 0.0002). **g** HeLa cells were treated with 30 μM NaAsO_2_ for 2 h and stress granules were observed by immunofluorescence staining of G3BP1 (red). The distribution of S6K1 and S6K2 were analysed by immunostaining (green). Yellow arrows indicate examples of stress granules. Nuclei were stained with DAPI (blue). Scale bar = 25 μm

Consistent with previous studies [22, 23], we observed the localisation of the mTORC1 associated proteins mTOR, RAPTOR and ASTRIN with the SG marker G3BP1 in HeLa cells treated with 0.5 mM sodium arsenite for 30 min to cause an acute oxidative stress (Fig. S1A-C). We also observed robust localisation of S6K2 to SGs with less pronounced co-localisation of S6K1 and the S6 kinase substrate RPS6 (Fig. 1a, S1D). Sodium arsenite treatment causes translational repression and SG formation via promoting the phosphorylation of Ser51 on eIF2α [27] but SGs also form independent of this following inhibition of eIF4A [6, 7]. We found that mTORC1 components, S6 kinases and RPS6 all localised to SGs following treatment with FL3, an eIF4A inhibitor [28, 29] (Fig. 1a, S1). As expected, FL3 suppressed translation without increasing eIF2α phosphorylation (Fig. S2). We conclude that S6K2, and to a lesser extent S6K1, are present in SGs formed by both eIF2α phosphorylation-dependent and independent mechanisms. The acute oxidative stress induced by high concentrations of sodium arsenite (e.g., 0.5 mM) may not be reflective of physiological conditions, so we investigated the formation of SGs under milder oxidative stress. Cells were treated with 30 μM sodium arsenite for up to 6 h and assessed for SG formation by immunostaining for the SG marker proteins G3BP1 and TIA1 (Fig. 1b). SG formation increased up to 2 h of treatment before declining (Fig. 1b–d) and this temporal profile correlated with the abundance of phosphorylated eIF2α (Fig. 1e). To determine how the changes in phospho-eIF2α levels were related to translational inhibition, we performed a puromycin incorporation assay [30]. Puromycin acts as an analogue of aminoacyl-tRNA and we found that its incorporation into newly synthesised proteins inversely correlated with phospho-eIF2α levels across the time course (Fig. 1f), indicating that mild oxidative stress promotes the phosphorylation of eIF2α coincident with decreased translation. Interestingly, cells subjected to 30 μM sodium arsenite for 24 h still displayed SGs (Fig. S3A), suggesting that SG formation may be oscillatory with a peak at 2 h, declining by 6 h, but with SGs re-appearing in most cells by 24 h. Importantly, we found that the SGs formed after mild arsenite stress have distinct characteristics compared to those induced by acute arsenite treatment. The aliphatic alcohol 1,6-hexanediol dissolves liquid structures by breaking weak hydrophobic bonds while leaving solid aggregates unaffected [14, 31], thus can distinguish between the liquid phase and solid aggregates within SGs. It is reported that high concentrations of this reagent induces SGs [18], so we used a concentration (0.5%) that we determined does not promote granule formation. Under these conditions, there was a decrease in the size of SGs formed after mild arsenite treatment but not after acute treatment (Fig. S4A-C), suggesting that SGs induced by mild oxidative stress contain a higher liquid composition. Further evidence that SGs formed after mild oxidative stress are distinct came from the use of ISRIB, which inhibits the actions of phosphorylated eIF2α [27]. SG formation under mild arsenite treatment was suppressed by ISRIB but this did not occur in response to the acute stress (Figs. S4D, E). Together, these data suggest that distinct types of SGs exist dependent on the level of oxidative stress. We found that S6K1 was significantly more abundant in SGs formed after mild arsenite treatment compared to those formed following acute arsenite stress, whilst S6K2 showed robust localisation to SGs in both conditions (Fig. 1a, g, S3B). Furthermore, the different isoforms of S6K1 and S6K2 [32, 33] all localised to SGs in response to mild arsenite treatment when ectopically expressed in HeLa cells (Fig. S5). Our data demonstrate that S6 kinases accumulate in distinct SGs formed following mild oxidative stress.

### S6K1 and S6K2 are required for SG formation

**Fig. 2:**
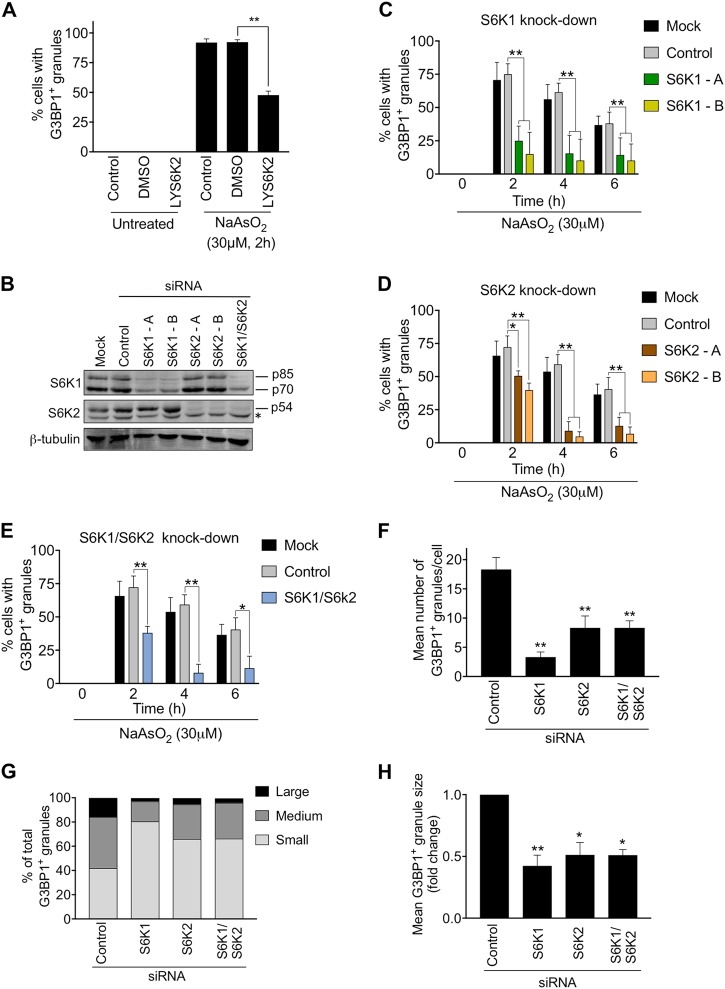
S6K1 and S6K2 are required for stress granule assembly. **a** HeLa cells were pre-treated or not with 0.5 mM LYS6K2 for 12 h and then treated with or without 30 μΜ of NaAsO_2_ for 2 h. Quantification of cells with G3BP1-positive granules under the indicated conditions is presented. 100 cells were analysed in each of the 3 biological repeats. Statistical analysis was carried out using one-way Anova (**p < 0.0002). **b** HeLa cells were transfected with siRNAs against S6K1 and S6K2 and the levels of knockdown were confirmed via immunoblotting of cell lysates. β-tubulin levels were analysed to ensure equal loading of lysates. A non-specific band in the S6K2 blot is indicated with a star (*). **c–e** Quantification of cells with stress granules after treatment with 30 μΜ of NaAsO_2_ for the indicated times and either siRNAs against S6K1 (S6K1-A and S6K1-B), S6K2 (S6K2-A and S6K2-B) or both S6K1 and S6K2 (S6K1-B and S6K2-B). 100 cells were analysed in each of the 4 biological repeats. Error bars are s.e.m. Data were analysed using two-way Anova (*p < 0.04; **p < 0.0002). **f** Mean number of granules per cell in those cells that displayed stress granules after 2 h treatment with 30 μΜ of NaAsO_2_. **g**, **h** Analysis of the size of stress granules following siRNA knockdown of S6K1 or S6K2 in cells treated for 2 h with 30 μΜ of NaAsO_2_. SGs were clustered into 3 groups according to their size: small, intermediate and large (see Materials and Methods) (**g**). Mean granule size was also measured (**h**). 100 cells were analysed in each of the 3 biological repeats. For **f** and **h**, error bars are s.e.m and data were analysed using one-way Anova (*p < 0.04; **p < 0.0002)

We next determined if S6 kinases played a role in regulating SG dynamics. Treatment with LYS6K2, an inhibitor of S6 kinase activity [34], caused a decrease in cells displaying SGs in response to mild arsenite stress (Fig. 2a, S6A, B). To confirm the importance of S6 kinases for SG assembly and investigate the specific roles of S6K1 and S6K2, their expression was knocked-down using siRNA (Fig. 2b, S6C). S6K1 depletion decreased SG number and size after mild arsenite treatment, as judged by immunostaining for the SG marker proteins G3BP1 and TIA1 (Fig. 2c, f–h, S7). S6K2 depletion caused a more modest decrease in the number of cells displaying SGs after 2 h of arsenite treatment (Fig. 2d, S7), but there were fewer granules per cell and they were smaller than in control cells (Fig. 2f–h, S7). At later time points of arsenite treatment, S6K2 depletion did significantly decrease the number of cells with SGs (Fig. 2d, S7C). The depletion of both S6K1 and S6K2 together did not produce an accumulative effect on SG formation or size (Fig. 2e–h). Importantly, the depletion of either S6K1 or S6K2 did not affect SG formation in response to acute arsenite stress (Fig. S8A-D). In conclusion, S6K1 and S6K2 are both required for SG assembly in response to mild oxidative stress. To determine if they also regulate SG assembly in response to other stimuli, we performed a similar analysis using heat stress and the eIF4A inhibitor, FL3. We found that S6K1 knockdown significantly decreased the number of cells with SGs and SG size in response to heat, while S6K2 knockdown only impacted on SG size (Fig. S9). In contrast, knockdown of S6K1 did not affect SG formation in response to FL3, while S6K2 knockdown reduced the number of cells displaying SGs as well as their size (Fig. S10). Our data suggest that S6 kinases play distinct roles in SG assembly dependent on the type of stress.

### S6K2 promotes SG persistence

**Fig. 3:**
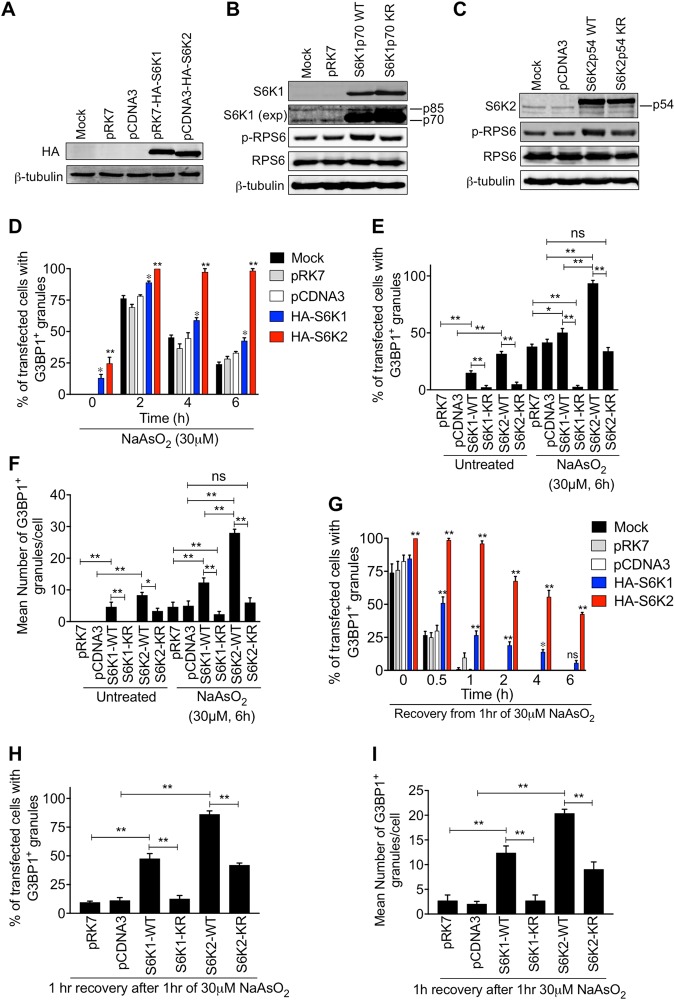
S6 kinases promote stress granule assembly and persistence dependent on their kinase activities. **a–c** Immunoblots of lysates from HeLa cells expressing HA-tagged S6K1p70 or S6K2p54 and corresponding kinase-inactive versions (KR). β-tubulin levels were analysed to ensure equal loading of lysates. Transfection with the parent vectors pRK7 and pCDNA3 was used as a control. Representative blots using antibodies against the HA-tag (**a**), S6K1 (**b**) and S6K2 (**c**) are shown. Comparison between the levels of ectopically expressed and endogenous S6K1 and S6K2 can be observed. The endogenous S6K1 (p85 and p70) can be observed in a longer exposure of the S6K1 blot (exp) and the endogenous S6K2 is labelled as p54. Levels of phosphorylated RPS6 (p-RPS6) and RPS6 are also shown. (**d**) HeLa cells expressing HA-S6K1p70 or HA-S6K2p54 were treated with 30 μM NaAsO_2_ for the indicated times and the % of cells with stress granules was quantified. **e**, **f** HeLa cells expressing HA-S6K1p70, HA-S6K2p54 or kinase-inactive mutants (KR) were treated with 30 μM NaAsO_2_ for 6 h. The % of cells with stress granules (**e**) and the mean number of granules in the cells that displayed stress granules were quantified (**f**). **g** HeLa cells expressing HA-tagged S6K1p70 or S6K2p54 were subjected to 30 μM NaAsO_2_ for 1 h and left to recover for the indicated times. The % of cells displaying stress granules was quantified. **h**, **i** HeLa cells expressing HA-S6K1p70, HA-S6K2p54 or kinase-inactive mutants (KR) were treated with 30 μM NaAsO_2_ for 1 h and left to recover for 1 h. The % of cells with stress granules (**h**) and the mean number of granules in the cells that displayed stress granules were quantified (**i**). All quantifications are from 100 cells per condition in each of the 3 biological repeats. Error bars are s.e.m. For **d** and **g**, statistical analysis was performed using two-way Anova and for **e**, **f**, **h** and **i** by one-way Anova (ns = not significant; *p < 0.04; **p < 0.0002)

Our observation that S6K2 knockdown caused only a partial reduction in the number of cells with SGs after 2 h of mild arsenite treatment, but a robust decrease after longer treatments (Fig. 2d), led us to speculate that it might play a more predominant role in maintaining SGs. To address this, we ectopically expressed S6K1p70 or S6K2p54 isoforms in cells (Fig. 3a–c). Even in the absence of arsenite treatment, around 10% of the S6K1-expressing cells and 25% of S6K2-expressing cells displayed SGs (Fig. 3d, S5F, S11). After 2 h of 30 μM arsenite treatment, around 75% of control cells formed SGs and this was enhanced in S6K1- and S6K2-expressing cells (Fig. 3d). However, the number of control and S6K1-expressing cells displaying SGs decreased at later time points, but SGs remained present in all the S6K2-expressing cells (Fig. 3d, S11). To determine if S6K2 could maintain SGs after stress removal, cells were treated with 30 μM arsenite for 1 h and left to recover. SGs persisted in the S6K2-expressing cells compared to the control cells in which the SGs rapidly dissolved (Fig. 3g). S6K1-expressing cells displayed a modest effect on maintaining SGs during the recovery period (Fig. 3g). These data suggest that S6K2 may play a major role in promoting the persistence of SGs.

### S6 kinases promote SG assembly dependent upon their kinase activity

The observations that mild oxidative stress and FL3 treatment both increase S6 kinase activity (Fig. S12) and that an S6 kinase inhibitor reduces SG formation (Fig. 2a) strongly suggests a key enzymatic role for S6 kinases in SG regulation. In support of this, ectopic expression of a kinase-inactive form of S6K1p70 did not support SG assembly or persistence in response to mild arsenite stress (Fig. 3e, f, h, i, S11). However, around 25% of cells expressing kinase-inactive S6K2 still displayed SGs, although the number in each cell was reduced (Fig. 3e, f, S11). Furthermore, SG persistence after arsenite withdrawal was only partially suppressed by kinase-inactive S6K2 (Fig. 3h, i, S11). This suggests that S6K2 may have both kinase-dependent and kinase-independent roles in SG regulation. We next investigated how the ectopic expression of the S6 kinases affected the response to FL3. We found that S6K2, but not S6K1, strongly promoted SG formation in the presence of FL3 and this was dependent on S6K2 kinase activity (Fig. S13). This supports our data from knockdown experiments demonstrating that S6K2, but not S6K1, can regulate SG assembly and persistence in response to eIF4A inhibition (Fig. S10). Taken together, these data suggest that the enzymatic activity of S6 kinases is important for promoting SG assembly and persistence.

### Signalling by mTORC1 promotes SG formation

**Fig. 4:**
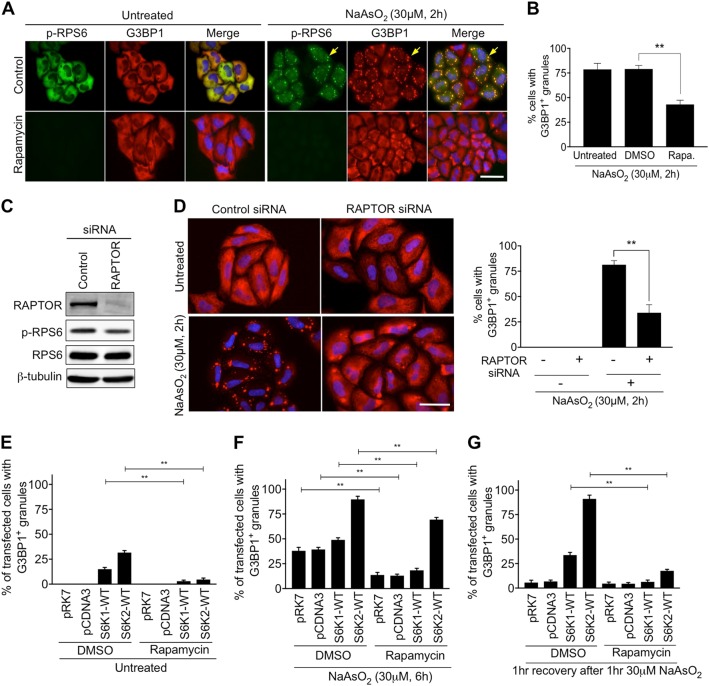
The mTORC1-S6K signalling pathway is required for stress granule assembly. **a** HeLa cells were pre-treated with 50 nM Rapamycin for 2 h prior to exposure to 30 μM NaAsO_2_ for a further 2 h. Cells were immunostained for G3BP1 (red) and the phosphorylated form of RPS6 (p-RPS6) (green). Nuclei were stained with DAPI (blue). Yellow arrows indicate examples of stress granules. Scale bar = 25 μm. **b** Quantification of cells forming stress granules under the indicated conditions. **c** HeLa cells were subjected to siRNA directed against RAPTOR. Immunoblots of cell extracts with antibodies to RAPTOR, phosphorylated RPS6 (p-RPS6), RPS6 and β-tubulin are shown. **d** Images of cells treated with or without 30 μM NaAsO_2_ for 2 h and subjected to RAPTOR siRNA. Cells were immunostained for G3BP1 (red). Nuclei were stained with DAPI (blue). Scale bar = 50 μm. Quantification of cells forming stress granules under the indicated conditions is presented. **e–g** Cells expressing S6K1p70 or S6K2p54 were pre-treated with DMSO or rapamycin for 2 h and then subjected to either 30 μM NaAsO_2_ for a further 6 h (**f**) or 30 μM NaAsO_2_ for 1 h and left to recover for 1 h (**g**). For all quantifications, 100 cells per condition were counted in each of the 3 biological repeats. Error bars are s.e.m. Data were analysed using one-way Anova (**p < 0.0002)

S6 kinases are downstream targets of mTORC1 and the treatment of cells with the mTORC1 inhibitor rapamycin or knockdown of the mTORC1 component RAPTOR decreased S6 kinase activity and the number of cells displaying SGs after mild arsenite treatment (Fig. 4a–d). Rapamycin also suppressed the ability of overexpressed S6K1p70 and S6K2p54 to promote SG assembly and persistence in untreated cells (Fig. 4e, S14). However, it did not affect SG assembly in response to acute arsenite treatment (Fig. S8E-H). In the presence of 30 μM arsenite for 6 h, a condition where the expression of S6K2 but not S6K1 promotes SG persistence (Fig. 3d), rapamycin caused only a partial decrease in the number of S6K2-expressing cells displaying SGs (Fig. 4f, S14). When arsenite stress was removed and cells allowed to recover, SG persistence was substantially decreased by rapamycin in the S6K2-expressing cells, but not to the level in control or S6K1-expressing cells (Fig. 4g, S14). These results suggest that there are mTORC1-dependent and independent roles for S6K2 in promoting SG persistence, consistent with our data indicating a potential kinase-independent function of S6K2 (Fig. 3e, h, i).

### The mTORC1-S6K pathway regulates eIF2α phosphorylation and translation initiation in response to mild oxidative stress

**Fig. 5:**
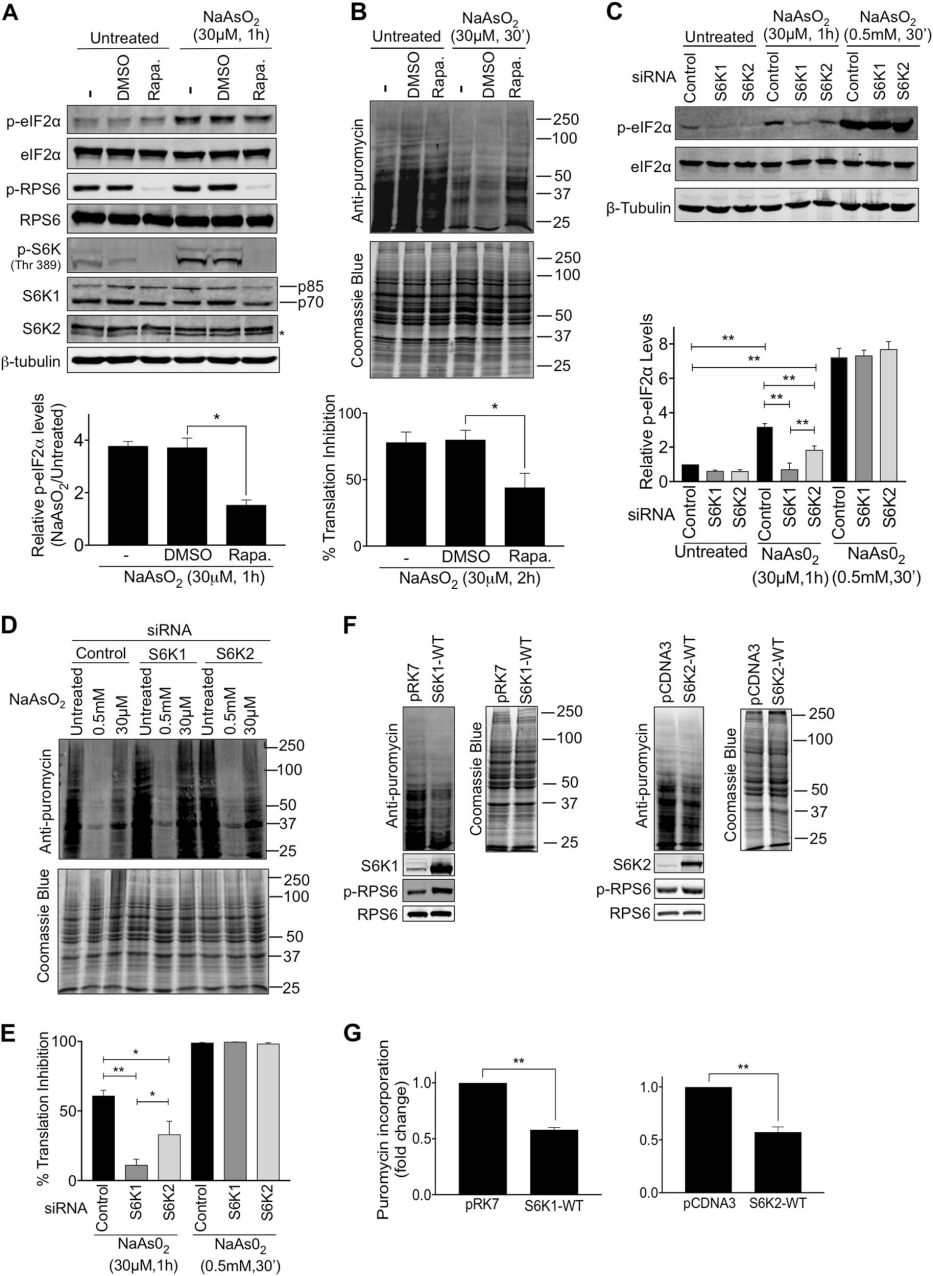
S6 kinases promote the phosphorylation of eIF2α and translation inhibition. **a** Immunoblotting of HeLa cell lysates for Ser51 phosphorylation on eIF2α (p-eIF2α) after treating cells with or without 30 μM NaAsO_2_ for 1 h in the presence of either DMSO or 50 nM rapamycin. Levels of phosphorylated RPS6 (p-RPS6) and S6 kinases (p-S6K) are shown to indicate the effectiveness of rapamycin at inhibiting mTORC1 activity. Blots for total eIF2α, RPS6, S6K1 and S6K2 protein levels are also presented. Quantification of p-eIF2α band intensities from 4 independent experiments is shown. The p-eIF2α levels were normalised against β-tubulin levels and are presented as relative increase in phosphorylation between untreated samples and those treated with NaAsO_2_. **b** HeLa cells were treated with 30 μΜ NaAsO_2_ for 30 min and the incorporation of puromycin into newly synthesised protein was assessed by immunoblotting. Band intensities for each lane were measured in 4 independent experiments and normalised against the intensity of Coomassie blue staining and presented as % translation inhibition. **c** HeLa cells transfected with siRNAs against S6K1 or S6K2 were treated with either 30 μΜ NaAsO_2_ for 1 h or 0.5 mM NaAsO_2_ for 30 min. Protein extracts were immunoblotted for p-eIF2α and total eIF2α protein levels. Quantification of p-eIF2α band intensities from 5 independent experiments is shown. The p-eIF2α levels were normalised against β-tubulin levels. **d**, **e** HeLa cells transfected with siRNAs against S6K1 or S6K2 were treated with either 30 μΜ NaAsO_2_ for 1 h or 0.5 mM NaAsO_2_ for 30 min and the incorporation of puromycin into newly synthesised protein was assessed by immunoblotting. Band intensities for each lane were measured in 3 independent experiments and normalised against the intensity of Coomassie blue staining and presented as % translation inhibition (**e**). **f**, **g** HeLa cells transfected with empty plasmids or plasmids expressing HA-S6K1p70 or HA-S6K2p54 were assessed for the incorporation of puromycin into newly synthesised protein by immunoblotting. The expression of the S6 kinases increased the levels of phosphorylated RPS6 (p-RPS6). Quantification of puromycin incorporation normalised against the intensity of Coomassie blue staining in 3 independent experiments is presented (**g**). Error bars are s.e.m and the data analysed using one-way Anova (*p < 0.04; **p < 0.0002)

Having established the importance of the mTORC1-S6K pathway for SG assembly and persistence, we investigated how this might occur. Both the inhibition of mTORC1 activity by rapamycin and the depletion of S6K1 or S6K2 led to reduced levels of eIF2α (Ser51) phosphorylation and suppression of the translation inhibition observed after mild arsenite treatment (Fig. 5a–e). Notably, S6K1 depletion caused a more significant suppression of arsenite-induced translation inhibition compared to S6K2 depletion (Fig. 5d, e), consistent with the greater suppression of eIF2α phosphorylation observed after S6K1 depletion (Fig. 5c). Acute arsenite stress led to a robust inhibition of translation that was not affected by depletion of either S6 kinase (Fig. 5c–e), providing further evidence that there are distinct signalling mechanisms promoting SG assembly dependent on the level of oxidative stress. In agreement with the S6 kinase knockdown experiments, ectopic expression of S6K1 or S6K2 increased eIF2α phosphorylation and partially suppressed translation (Fig. 5f, g, S15). Taken together, our findings indicate that the mTORC1-S6K pathway may promote SG assembly in response to mild oxidative stress via eIF2α phosphorylation. To explore this further, we analysed whether ISRIB further exacerbated the suppression of SG assembly that occurs following S6 kinase knockdown. ISRIB did not further reduce SG formation following S6K1 depletion (Fig. S16), indicative of S6K1 and eIF2α acting in the same pathway. However, ISRIB did enhance the reduction in SG assembly caused by S6K2 depletion (Fig. S16), suggesting that S6K2 contributes to SG assembly via eIF2α phosphorylation and additional mechanisms.

### The *C. elegans* S6 kinase orthologue, RSKS-1, promotes assembly of SGs

**Fig. 6:**
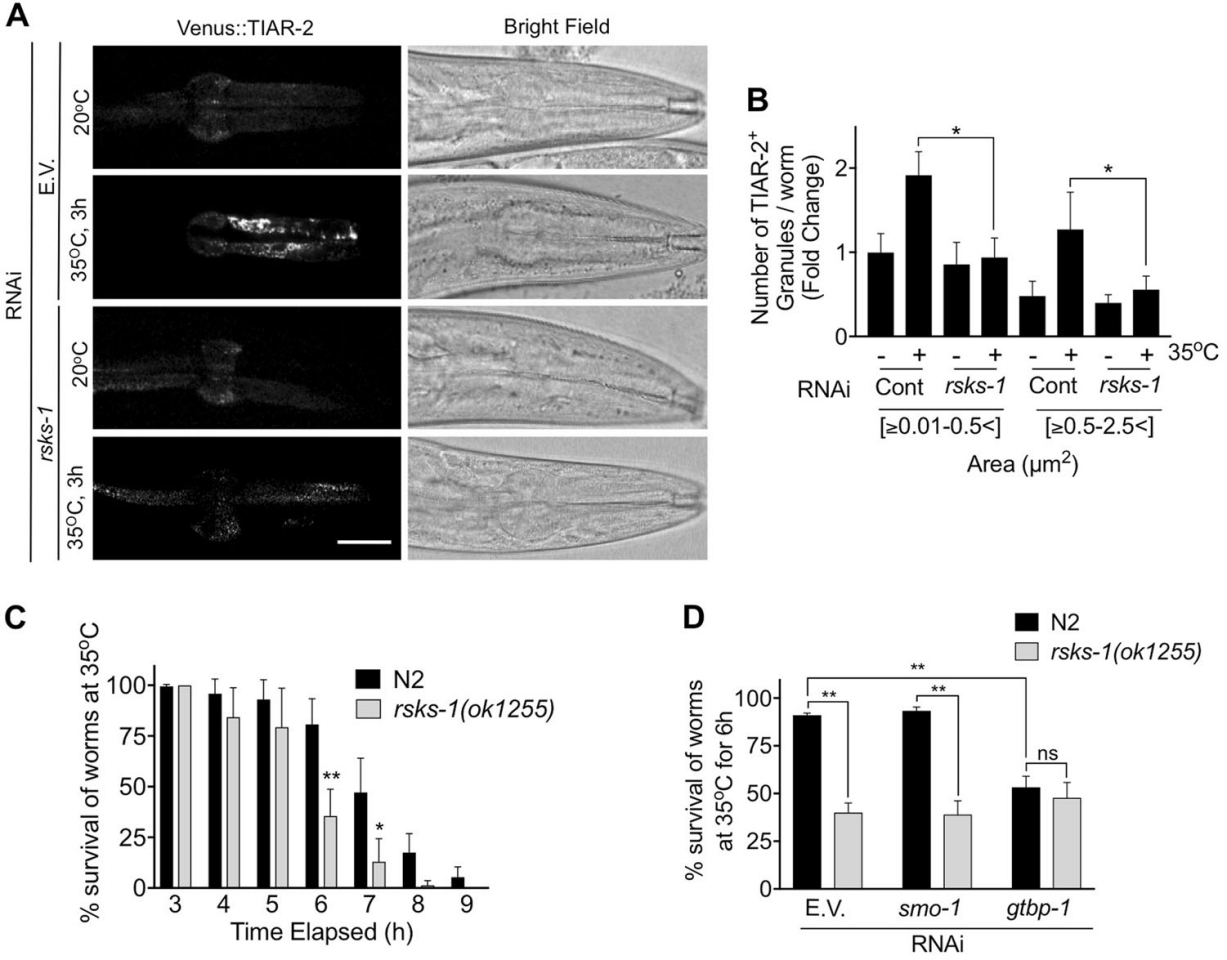
The *C. elegans* S6 kinase orthologue, RSKS-1, promotes stress granule formation in vivo. **a** Worms expressing a pharyngeal Venus::TIAR-2 reporter were fed *rsks-1* RNAi and subjected to heat shock at 35 °C for 3 h. Images of the pharynx of worms are shown for the indicated conditions. Left-hand panels show reporter expression and the right-hand panels are corresponding bright field images. Scale bar = 25 μM. **b** Quantification of the number of TIAR-2 positive granules separated by size, either small (0.01–0.5 μm^2^) or large (0.5–2.5 μm^2^). 30 worms were analysed per condition in 3 biological repeats and data analysed by two-way Anova (ns = not significant; *p < 0.04). **c** Worm survival assay. N2 or *rsks-1(ok1255)* mutant worms were subjected to heat shock at 35 °C and the number of worms alive at the indicated times was scored. At least 30 worms were assessed in each of the 3 biological repeats and data were analysed using two-way Anova (*p < 0.04; **p < 0.0002). **d** Epistasis experiment to determine the relationship between *rsks-1* and *gtbp-1*. N2 and *rsks-1(ok1255)* worms were fed with *gtbp-1(RNAi)* and subjected to heat stress (35 °C for 6 h). Worm survival was recorded. The *smo-1* RNAi was used as a negative control as it does not suppress stress granule assembly. 30 worms were analysed per condition in 3 biological repeats and data were analysed using one-way Anova (ns = not significant; **p < 0.0002)

Many studies have focussed on the function of SGs in cultured cells, but their role in metazoan stress responses is poorly characterised. Therefore, it was important to determine if S6 kinases were required for SG assembly *in vivo*. SGs have been characterised in the nematode worm *C. elegans* and shown to regulate their response to stress [35–38]. *C. elegans* express a single S6 kinase orthologue, RSKS-1, which we targeted with RNAi. We utlised a reporter strain featuring pharyngeal muscle expression of the SG protein TIAR-2 fused to the fluorescent protein Venus [38]. Previous studies have demonstrated robust formation of SGs in *C. elegans* in response to heat stress [37, 38], a condition that requires S6 kinases for optimal SG assembly in HeLa cells (Fig. S9). We therefore determined the effect of the *rsks-1(RNAi)* on SG formation in *C. elegans* subjected to heat stress. The number and size of TIAR-2 positive granules increased upon heat stress in wild-type nematodes but not after RSKS-1 knockdown (Fig. 6a, b), indicating the requirement for RSKS-1. Furthermore, we employed a loss-of-function strain [39] to demonstrate that RSKS-1 was important for survival in response to heat stress (Fig. 6c), consistent with a previous study [40]. To determine if the increased sensitivity of the *rsks-1* mutant worms is due to their inability to efficiently form SGs, we performed an epistasis assay with *gtbp-1*, the orthologue of mamamlian *G3BP1*. As in mammalian cells, GTBP-1 is required for SG assembly (Fig. S17). Consistent with a role for SGs in protecting *C. elegans* from heat stress, we found that *gtbp-1(RNAi)* reduced *C. elegans* survival by around 50% (Fig. 6d). However, when knockdown of GTBP-1 was performed in the *rsks-1* mutant, survival was not further decreased (Fig. 6d). This suggests that RSKS-1 and GTBP-1 are likely to be acting in the same pathway required for SG assembly. Taken together, these data indicate that promotion of SG formation by S6 kinase plays a role in *C. elegans* survival during heat stress and are supportive of S6 kinases being evolutionarily conserved mediators of SG assembly.

## Discussion

Recent studies have indicated a complex relationship between mTOR signalling and SGs in mediating cellular responses to stress. mTORC1 components accumulate in SGs leading to suppression of mTORC1 signalling [21–23] whilst, on the other hand, there is evidence that mTORC1 signalling may be important for the formation and maintenance of SGs [25, 26]. Here, we show that the mTORC1 effector kinases S6K1 and S6K2 play important roles in SG assembly and persistence in human cells and that the S6 kinase orthologue RSKS-1 is required for robust SG formation and protection from heat stress in *C. elegans*.

**Fig. 7:**
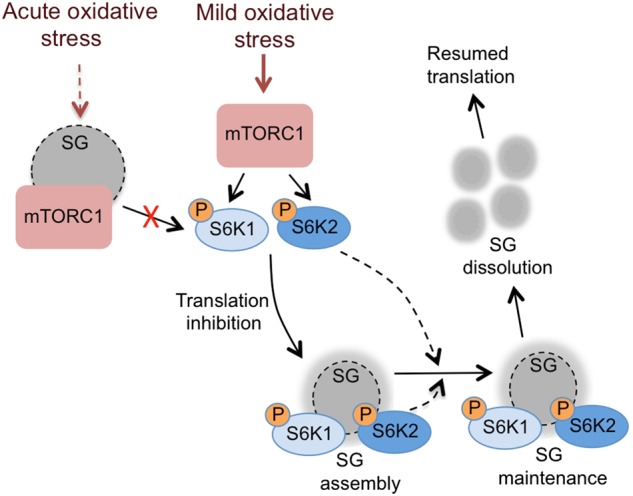
Schematic of the role of the mTORC1-S6 kinase pathway in stress granule assembly and maintenance. Distinct types of stress granule (SG) are formed depending on the level of oxidative stress. In response to acute oxidative stress, mTORC1 components are sequestered into solid SGs thus inhibiting mTORC1 activity [22, 23]. In response to mild oxidative stress, mTORC1 activates S6 kinases leading to inhibition of translation and the assembly of SGs that contain a solid core surrounded by a liquid shell. Both S6K1 and S6K2 can accumulate in SGs and S6K2 helps maintain SGs via an unknown mechanism. On recovery from the stress, the SGs dissolve and protein translation resumes

We demonstrate that S6K1 and S6K2 are novel components of SGs (Fig. 1) and this is supported by a recent report showing that ectopically expressed S6K2 is present in cytoplasmic granules [41]. Both kinases play a regulatory role in SG assembly in response to mild oxidative stress but not in response to acute stress (Figs. 2 and 3, S8). This is consistent with the distinct properties of SGs under these different conditions, with those formed after mild oxidative stress being more dependent on the actions of phosphorylated eIF2α and containing a higher liquid content (Fig. 5, S4). Importantly, the roles of S6K1 and S6K2 in SG regulation are distinct. The initial assembly of SGs in response to mild oxidative stress appears more dependent on S6K1, whilst S6K2 plays a more predominant role in SG persistence (Figs. 2, 3 and 7) and has both kinase-dependent and kinase-independent functions in SG regulation (Figs. 3 and 4f, g). The knockdown of both S6 kinases together did not further impact on SG formation or persistence (Fig. 2e), possibly due to this causing a more severe translational inhibition that promotes some SG assembly independently of S6 kinases (as occurs in response to high levels of arsenite stress). S6K1, and to a lesser extent S6K2, regulate eIF2α phosphorylation at Ser51 and the suppression of translation (Fig. 5c–e, S16). HRI kinase is required for eIF2α phosphorylation in response to arsenite [5, 42], so it is possible that S6 kinases promote HRI activity. Alternatively, they may regulate phosphatases that target eIF2α [43, 44], or promote the binding of eIF2B to phosphorylated eIF2α, by protecting it from de-phosphorylation. Whilst we observe a correlation between eIF2α phosphorylation and translation inhibition, other S6 kinase-dependent mechanisms may also be important. For example, it is reported that the phosphorylation of RPS6 by S6 kinases can negatively affect global translation [45], so the reduction in RPS6 phosphorylation caused by the knockdown of S6 kinases could contribute to the suppression of the translation inhibition observed in response to mild oxidative stress.

The relative importance of the S6K1 and S6K2 can vary depending on the form of stress. We found that S6K1 plays a more dominant role than S6K2 in SG assembly in response to heat stress (Fig. S9) but it is not required for SG formation following inhibition of eIF4A, which is independent of eIF2α phosphorylation (Fig. S10). This supports the case that S6K1 regulates eIF2α phosphorylation to promote SG assembly whilst S6K2 has additional actions, indicative of a broader function in SG assembly and persistence. While the precise mechanisms by which S6 kinases regulate SGs remain to be elucidated, their localisation to SGs suggests they could directly target other SG components. The fluid shell of SGs may provide an environment that promotes specific enzymatic reactions, as well as enhancing protein–protein interactions [17]. Interestingly, S6K2 is reported to phosphorylate hnRNPA1, which re-localises from nuclei to SGs upon arsenite stress [46, 47]. As S6K2 also localises to nuclei (Fig. 1g), it is possible that it may translocate with hnRNPA1 to SGs and they act together to regulate SG assembly or persistence. Such a co-dependency between the protein kinase RSK2 and the SG protein TIA1 in SG assembly has previously been proposed [20].

Overall, our data support an important role for mTORC1 effectors in promoting SG formation and functioning and complements previous research showing that mTORC1 phosphorylation of 4EBP1 was necessary for formation of SGs [25]. However, an important question is how to rationalise the requirement of mTORC1 effectors for SG assembly with the observation that SGs inhibit mTORC1 signalling (Fig. 7) [22, 23]. A possibility is that S6 kinases and 4EBP1 are important for SG assembly and persistence under mild stress conditions, but once the level of stress reaches a certain threshold, then mTORC1 signalling is inhibited to prevent its hyperactivation that can lead to apoptosis. The complex relationship between mTORC1 signalling and SGs may be mirrored in other stress-regulated pathways. For example, the JNK MAP kinase pathway promotes SG formation [48], but JNK activity and apoptosis can be suppressed by the sequestration of the upstream kinase MEKK4 into SGs [19].

SGs are part of a conserved adaptive mechanism to promote survival in response to stressful conditions [3, 4]. Using the multicellular organism *C. elegans*, we demonstrated that S6 kinase is important for heat-induced SG formation and that this correlates with stress resistance (Fig. 6, S17). Interestingly, the dynamic nature of SGs becomes more restricted with age in *C. elegans*, with the formation of solid aggregates that correlate with decreased fitness [38]. Genetic interventions that restore SG dynamics lead to increased lifespan [38]. Although *rsks-1* mutants have increased sensitivity to stress, they display increased lifespan [39, 40], so it is possible RSKS-1 may contribute to the formation of solid SG aggregates during ageing. There is also increasing evidence that SG proteins are important components of aggregates that underpin a number of neurodegenerative disorders [49] and that enhanced mTORC1/S6 kinase signalling contributes to this [50]. Aberrant regulation of S6 kinases and SGs may also be relevant to other pathologies, including cancer [51–53].

## Materials and methods

### Cell culture and SG induction

HeLa cells (from ATCC) were cultured in Dulbecco’s Modified Eagles Medium (D6429, Gibco® Life Technologies) supplemented with 10% foetal bovine serum (FBS) (S181H, Biowest) and 1% penicillin–streptomycin solution (P4333, Sigma). Cells were grown at 37 °C in 5% CO_2_. For immunoblotting and immunofluorescence assays, 100,000 cells/well were added to 6-well dishes. For immunofluorescence experiments, cells were grown on cover slips. SGs were induced by addition of NaAsO_2_ or FL3 at the concentrations indicated in the figure legends. Heat stress was applied by incubating cells at 42 °C for 1.5 h. Rapamycin (R0395) and ISRIB (SML0843) were obtained from Sigma-Aldrich. LYS6K2 (ab146199) was from Abcam.

### Transfection of plasmids and siRNAs

Plasmids were transfected into cells using JetPEI® (101B-010N, Polyplus Transfections) and siRNAs using Lipofectamine® RNAiMAX Transfection Reagent (13778150, Thermofisher Scientific). Cells were incubated overnight in the presence of plasmid or siRNA and the medium was then changed and cells incubated a further 24 h prior to analysis. Plasmids pRK7-HA-S6K1-WT (#8984), pRK7-HA-S6K1-KR (#8985), pcDNA3-HA-S6K2p54-WT (#17729) and pcDNA3-HA-S6K2p54-KR (#17730) were obtained from Addgene. Plasmids pcDNA3-HA-S6K1p70, pcDNA3-HA-S6K1p85 and pcDNA3-HA-S6K2p56 were constructed by the Whitmarsh lab (details on request). Control siRNA (D-001810-02-05 and D-001810-02-05), S6K2 siRNA-B (L-004671-00-0005) and RAPTOR siRNA (J-004107-05-0002) were from Dharmacon. S6K1 siRNA-A (SI00301721), S6K1 siRNA-B (SI00048608) and S6K2 siRNA-A (SI00288120) were from Qiagen.

### Immunoblotting

Cells were washed with PBS and lysed in Triton lysis buffer (TLB) containing 20 mM Tris-HCl pH 7.4, 137 mM NaCl, 2 mM EDTA, 1% Triton X-100, 25 mM β-glycerophosphate, 1 mM Na_3_Va_4_, 10% glycerol, 1 mM phenylmethylsulphonyl fluoride (PMSF), 10 μg/ml aprotinin and 10 μg/ml leupeptin. Protein amount was quantified using the Pierce™ BCA Protein Assay Kit (Thermo Fisher Scientific) according to manufacturer’s protocol. About 25 μg of protein lysate was loaded on 10% polyacrylamide gels and electrophoresis performed at 130 V for 1.5 h. Resolved proteins were transferred to polyvinylidene difluoride membrane (PVDF, Millipore) using a semidry procedure (15 V for 2 h). Membranes were blocked with 5% BSA in TBST (Tris-buffered saline, 0.2% Tween-20) and incubated with primary antibodies overnight at 4 °C. Membranes were washed in TBST buffer and incubated for 1 h at room temperature with secondary antibody diluted in 5% BSA-TBST supplemented with 0.02% SDS, prior to washing with TBST buffer and imaging using the Odyssey infrared system (LI-COR Biosciences). Antibodies used are described in Table S1. For phospho-Ser51 eIF2α blots, blocking was performed with 5% milk-TBST at 4 °C overnight and membranes incubated with primary antibody diluted in 5% milk-TBST at room temperature for 2 h, prior to washing with TBST and incubation with secondary antibody in 5% milk-TBST supplemented with 0.02% SDS. Unprocessed scans of all immunoblots are provided in Figures S19 and S20.

### Puromycin incorporation assay

Performed as described [30]. Puromycin was added to cells for 5 min at a final concentration of 5 μg/ml prior to harvesting and lysis. The anti-Puromycin antibody (see Table S1 for details) was used at a dilution of 1:25,000.

### Immunofluorescence microscopy

Cells were fixed in 4% paraformaldehyde for 15 min at room temperature and permeabilised with PBS buffer containing 0.2% Triton X-100 (PBST) for 20 min. Cells were blocked with 3% BSA in PBST for 30 min and incubated overnight at 4 °C with the relevant primary antibodies (see Table S1). Following washes with TBST, cells were incubated in 3% BSA in PBST solution containing the relevant secondary antibody for 1 h at room temperature, before further washes in PBST and mounting using the ProLong® Diamond–DAPI (Life Technologies). Imaging was performed with a Leica DM500 B Fluorescence microscope with filter sets for DAPI, FITC and Texas Red. Images were collected with a Leica DCF340 FX camera at a magnification of 60× and an exposure of 100 ms. Images were processed with Image J software. A threshold was applied at a value of 142, images shifted to binary and the granules outlined. According to the size of the granules, three groups were identified: small: 0.01–0.6 μm^2^, medium: 0.61–2 μm^2^ and large: 2.01–5 μm^2^. The mean granule size was also recorded. For co-localisation studies, Pearson’s correlation coefficient analysis was performed; 50 cells were analysed in ImageJ for each condition. The co-localisation was assessed using coloc 2. The data are presented in Figure S18.

### *C. elegans* strains, maintenance and synchronisation

Strains were grown at 20 °C on nematode growth media (NGM, 50 mM NaCl, 0.25% (w/v) Bacto-Peptone, 1.7% (w/v) Agar) supplemented with CaCl_2_ (1 mM), MgSO_4_ (1 mM), KH_2_PO_4_ (25 mM), Cholesterol (5 µg/ml) spotted with OP50 *Escherichia coli* [54]. Strains used: Bristol N2; *rsks-1(ok1255)* RB1206 which is a loss-of-function strain [39] resulting from the deletion of 1700 bp in exon 4 (obtained from the *Caenorhabditis* Genetics Center); DCD194: N2; *uqEx41[Pmyo-2::venus::tiar-2]* provided by Della David (German Center for Neurodegenerative Diseases, Tubingen) [38]. To acquire a synchronised population, gravid adults were washed in 15 ml falcon tubes with M9 buffer (42 mM Na_2_HPO_4_·12H_2_O, 22 mM KH_2_PO_4_, 86 mM NaCl, 1 mM MgSO_4_) and then incubated in 1 ml bleaching solution for 3–4 min (8 M NaOH, 20% (v/v) sodium hypochlorite). Resulting embryos were washed in M9 three times and seeded on NGM plates without food.

### *C. elegans* RNAi

RNAi by feeding was carried out as previously described [55]. Briefly, NGM plates were supplemented with Carbenicillin (50 µg/ml), IPTG (1 mM) and Nystatin (50 U/ml). Worms were randomly allocated to either control plates with HT115 (DE3) *E. coli* bacteria transformed with empty L4440 plasmid vector or to plates with HT115 (DE3) *E. coli* expressing RNAi targeting *rsks-1* (ORF-ID Y47D3A.16) and *gtbp-1* (ORF-ID K08F4.2) (purchased from the Vidal library of ORF-RNAi available at SourceBioScience). RNAi targeting *smo-1* (ORF-ID K12C11.2), the *C. elegans* orthologue of mammalian *SUMO1*, was obtained from the Ahringer RNAi library [55] and used as a negative control. Its efficacy was checked by observing known *smo-1* RNAi phenotypes including low brood size [56]. RNAi vectors were fed to synchronised L4 worms and the F1 generation kept on RNAi until analysis at day 1 of adulthood. The nematodes were placed at 35 °C for 3 h and SG formation was imaged immediately afterwards.

### *C. elegans* imaging

For live imaging, worms were paralysed in 20 mM tetramisole for 2 min before mounting onto agarose pads. A Leica DM500 B Fluorescence microscope was used with filter sets for DAPI and FITC. Images were collected with a Leica DCF340 FX camera at a magnification of 60× and an exposure of 100 ms. Images were processed with Image J software. A threshold was applied between values of 130–50, images shifted to binary and the granules outlined. The representative images presented here were obtained using a Leica TCS SP5 AOBS inverted confocal using a 63×/0.6–1.40 Plan Fluotar objective. The confocal settings were as follows, pinhole [1 airy unit], scan speed [1000 Hz unidirectional] and format [512 × 512] or [1024 × 1024]. Images were collected using PMT detectors with the following detection mirror settings; FITC 494–530 nm using the 488 nm (20%) laser line.

### Survival assay

*C. elegans* strains were synchronised at day 1 of adulthood and then moved to 35 °C for the indicated times. Worm viability was assessed by gentle prodding with a platinum wire. At each time point, the total number of worms (alive and dead) was counted and living worms were expressed as a percentage of the total number of worms.

### Statistical analysis

For cell immunofluorescence experiments, statistical analysis was carried out on 3 biological repeats unless otherwise stated. In each repeat, either 50 or 100 cells were counted per sample as stated in figure legends. The analysis was carried out using PRISM software and ordinary one- or two-way Anova. For *C. elegans* granule size measurements, 30 worms were imaged for the *rsks-1* RNAi and 20 worms for the *gtbp-1* RNAi and analysed using imageJ. The comparative analysis was carried out using ordinary one-way Anova. For the survival assay, at least 30 worms were counted at each time point in each of the 3 biological repeats and the results were analysed by two-way Anova. Error bars are s.e.m and variances are similar between the groups being compared and conform to a normal distribution. No statistical method was used to determine the sample sizes. Cells or worms were only excluded from analysis if the images were of insufficient quality.

## Electronic supplementary material

Supplemental Material
