# Correction to: The mTOR-S6 kinase pathway promotes stress granule assembly

**DOI:** 10.1038/s41418-019-0438-y

**Published:** 2019-10-22

**Authors:** Aristeidis P. Sfakianos, Laura E. Mellor, Yoke Fei Pang, Paraskevi Kritsiligkou, Hope Needs, Hussein Abou-Hamdan, Laurent Désaubry, Gino B. Poulin, Mark P. Ashe, Alan J. Whitmarsh

**Affiliations:** 1 School of Biological Sciences, Faculty of Biology, Medicine and Health, University of Manchester, Manchester Academic Health Science Centre, Michael Smith Building, Oxford Road, Manchester, M13 9PT UK; 2 Laboratory of Therapeutic Innovation (UMR 7200), CNRS, University of Strasbourg, 67401 Illkirch, France

**Keywords:** Cell biology, Genetics

**Correction to: Cell Death & Differentiation**

10.1038/s41418-018-0076-9

The original PDF version of this article incorrectly showed the copyright holder to be ‘ADMC Associazione Differenziamento e Morte Cellulare 2018’, when the correct copyright holder is ‘The Authors 2018’. This has been corrected in the PDF version of the article. The HTML version was correct from the time of publication.

