# Supplementary material for: The mTOR-S6 kinase pathway promotes stress granule assembly
Source: Cell Death Differ. 2018 Mar 9;25(10):1766–80. doi: 10.1038/s41418-018-0076-9 (PMC6004310; doi:10.1038/s41418-018-0076-9)
Supplement: Supplementary file 1 — Supplemental Material [file 41418_2018_76_MOESM1_ESM.pdf]

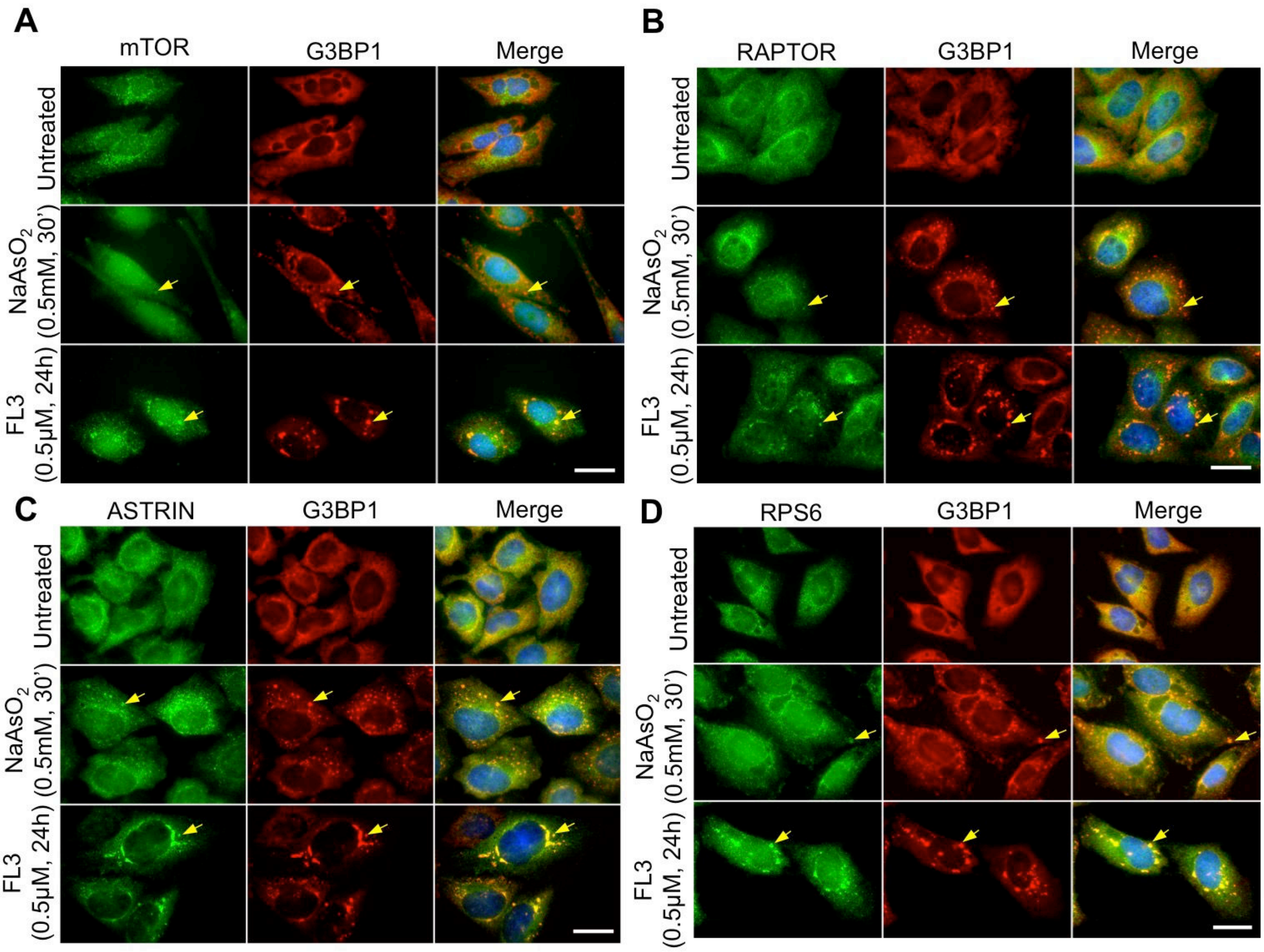

Figure S1: Components of the mTORC1 pathway localise to stress granules following arsenite stress or FL3 treatment. HeLa cells were treated with either 0.5mM of $NaAsO_2$ for 30 minutes or 0.5µM FL3 for 24 hours and stress granules labelled using an antibody against G3BP1 (red). The localisation of mTOR (A), RAPTOR (B), ASTRIN (C) and RPS6 (D) were observed by immunofluorescence staining (green). Nuclei were stained with DAPI (blue). Merged images are shown. Yellow arrows indicate examples of stress granules. Scale bar = 25µm.

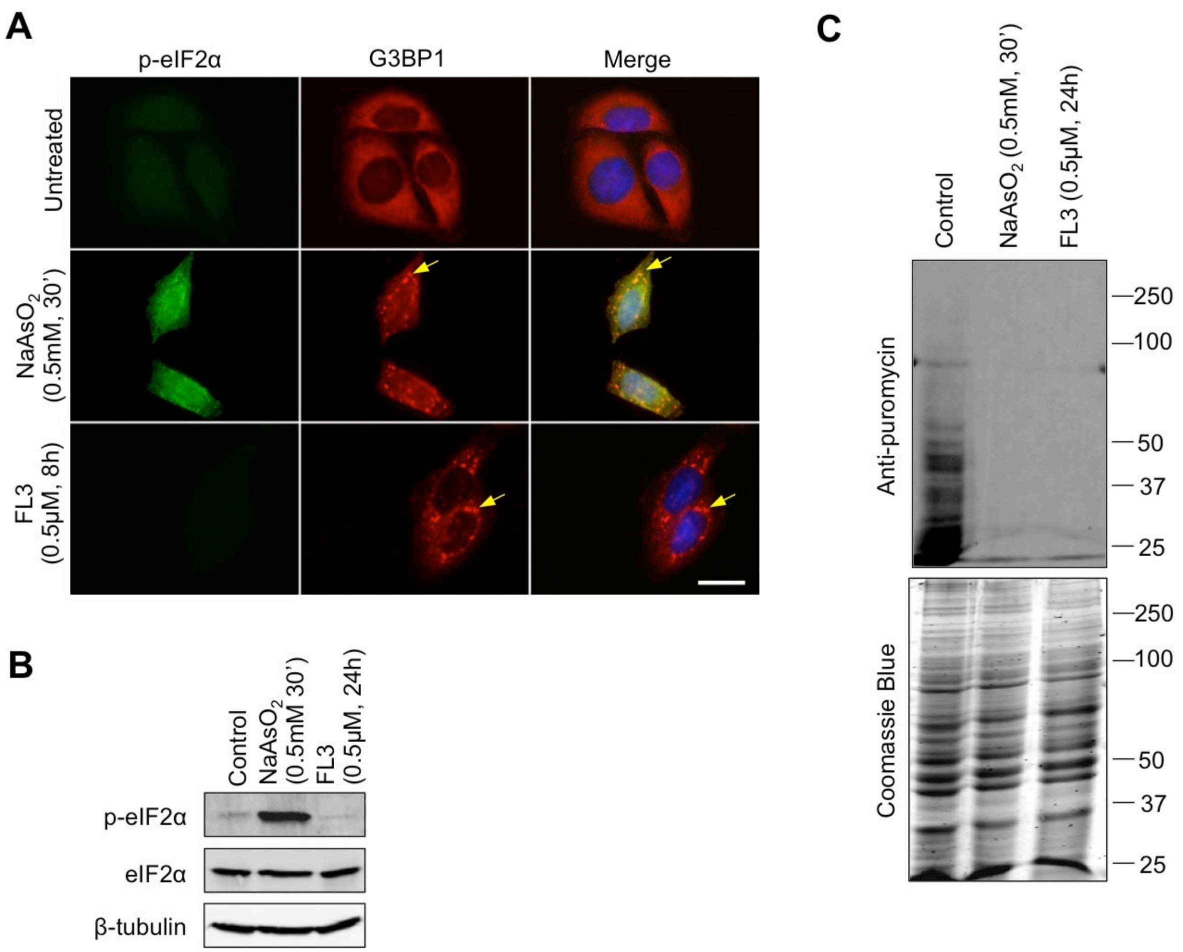

Figure S2: FL3 induces stress granules independently of eIF2α Ser51 phosphorylation. (**A**) HeLa cells were treated with either 0.5mM $NaAsO_2$ for 30 minutes or 0.5μM FL3 for 8 hours and the distribution of G3BP1 (red) and Ser51 phosphorylated eIF2α (p-eIF2α) (green) were observed by immunostaining. Nuclei were stained with DAPI (blue). Yellow arrows indicate examples of stress granules. Scale bar = 25μm. (**B**) Phosphorylated eIF2α (p-eIF2α) levels under the indicated conditions were determined by immunoblotting. β-tubulin levels were analysed to ensure equal loading of protein extracts. (**C**) Translation levels were investigated by ribopuromycilation assay and the incorporation of puromycin into newly synthesised protein assessed by immunoblotting. Coomassie blue staining of cell extracts was performed to show total protein amounts. Molecular weights (kDa) are indicated.

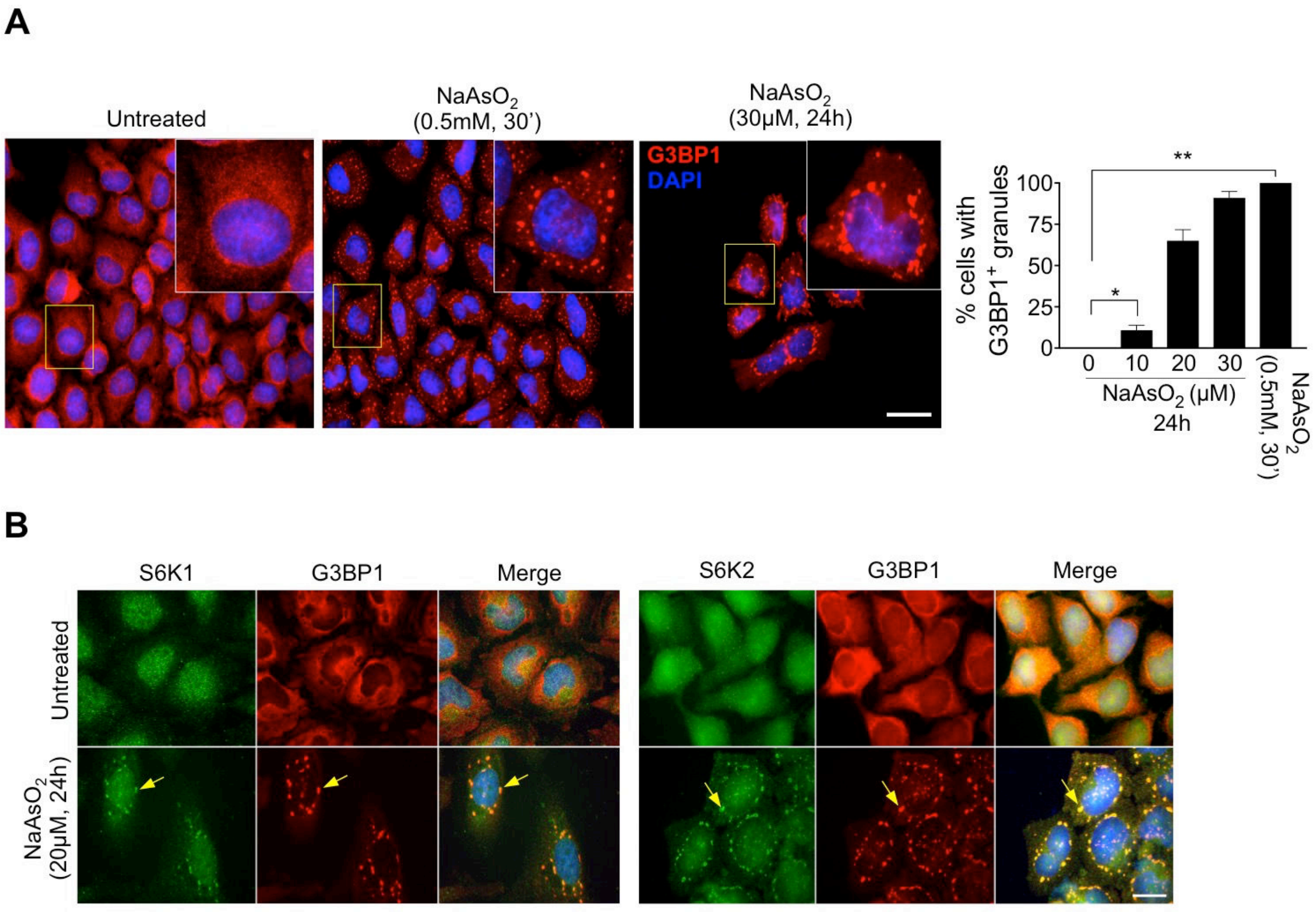

Figure S3: S6K1 and S6K2 localise to stress granules after long-term mild arsenite treatment. (**A**) HeLa cells were treated with either 0.5mM of NaAsO2 for 30 minutes or 10μM, 20μM or 30μM $NaAsO_2$ for 24 hours. Stress granule formation was assessed by immunofluorescence staining of G3BP1 (red). The insets in the upper right of each image are magnifications of the areas indicated by yellow boxes. Representative images and quantifications are shown. 50 cells were counted in each of 4 biological repeats. Error bars are s.e.m. Data analysed by one-way Anova (*p<0.04; **p<0.0002). (**B**) Cells treated with 20μM $NaAsO_2$ for 24 hours were imaged by immunofluorescent staining of S6K proteins (green) and G3BP1 (red). Nuclei were stained with DAPI (blue). Yellow arrows indicate examples of stress granules. Scale bars = 25μm.

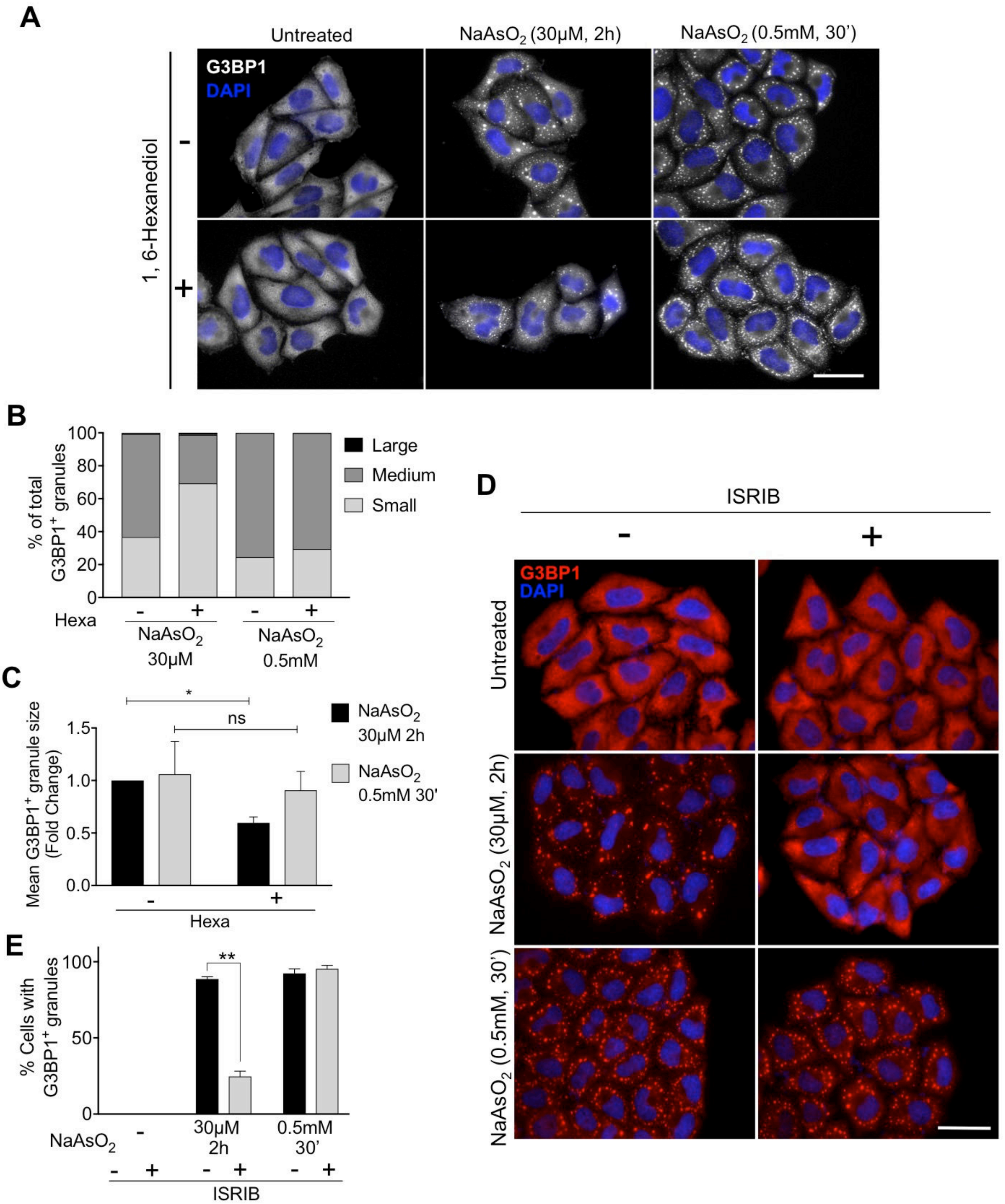

Figure S4: Stress granules have distinct biophysical and molecular properties dependent on the level of oxidative stress. (**A**) Cells were subjected to either 30μM of $NaAsO_2$ for 2 hours or 0.5mM $NaAsO_2$ for 30 mins prior to treatment with 0.5% 1,6-Hexanediol (Hexa) for 20 mins. Stress granules were observed by G3BP1 immunostaining (grey). Nuclei were stained with DAPI (blue). Scale bar = 50μm. (**B**) Stress granules formed after the indicated treatments were divided into 3 groups according to their size: small, intermediate and large (see Materials and Methods). (**C**) Mean granule size. 100 cells were analysed in each of the 4 biological repeats and statistical analysis was carried out using one-way Anova (ns=not significant; *p<0.04). (**D, E**) HeLa cells were treated with 200nM of ISRIB for 2 hours and either mild (30μM $NaAsO_2$ for 2 hrs) or acute (0.5mM NaAsO2 for 30 mins) oxidative stress. Stress granules were observed by immunostaining for G3BP1 (red). DNA was stained with DAPI (blue). Representative images and quantification of the number of cells with stress granules are shown. 50 cells were counted in each of 3 biological repeats. Error bars are s.e.m. Statistical analysis was carried out using one-way Anova (**p<0.0002). Scale bar = 50μm.

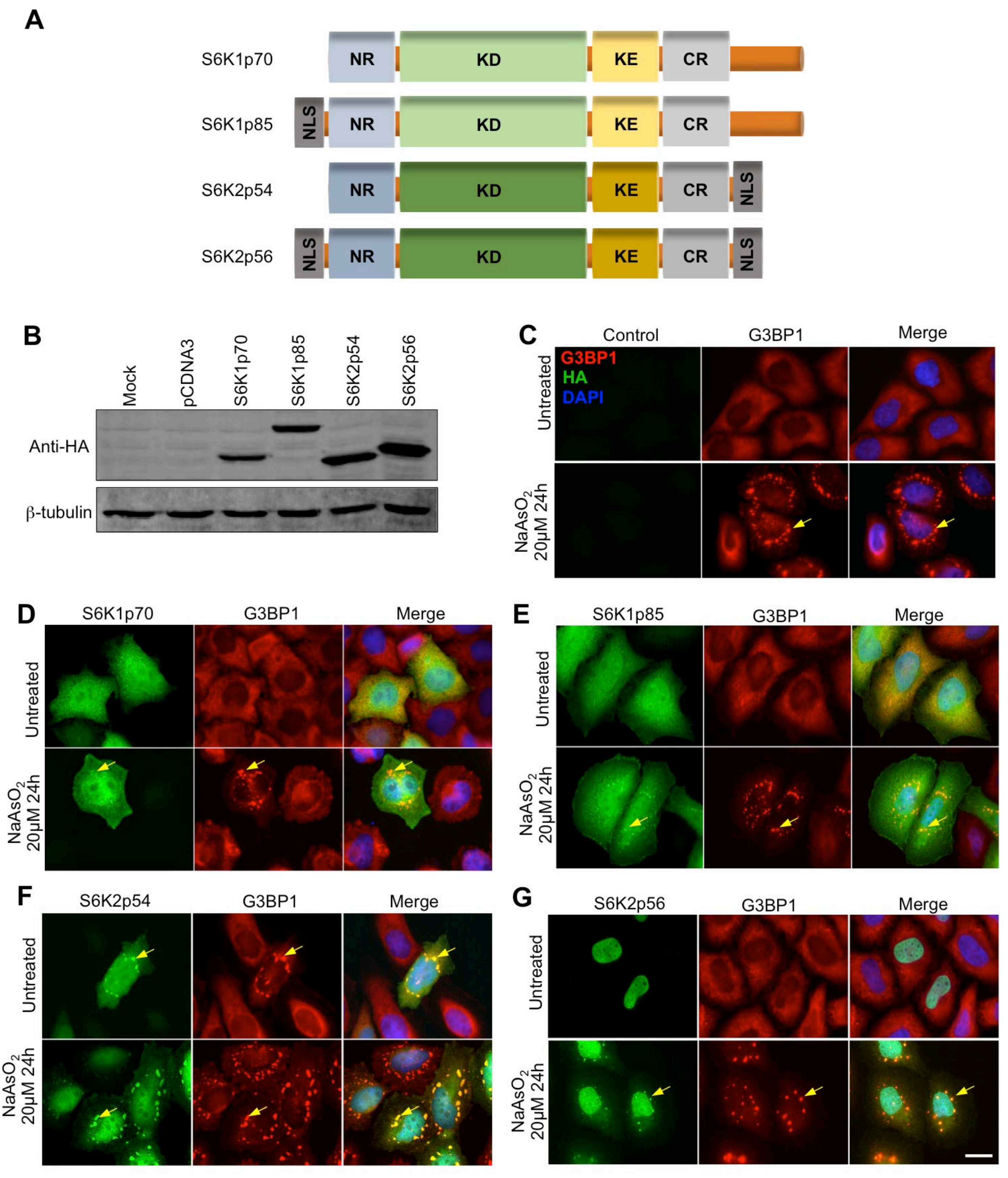

Figure S5: Localisation of distinct S6K1 and S6K2 isoforms to stress granules. (**A**) Representation of the domain structures of S6K1 and S6K2 isoforms. N=N-terminal domain, KD=kinase domain, KE=kinase extension domain, CR=C-terminal domain, NLS=nuclear localisation signal. (**B**) Expression levels of HA-tagged S6K1 and S6K2 isoforms determined by immunoblotting. Untransfected cells (Mock) and cells transfected with empty vector (pCDNA3) were used as controls. β-tubulin levels were analysed to ensure equal loading of lysates. (**C-G**) HeLa cells expressing HA-tagged S6K1p70, S6K1p85, S6K2p54 and S6K2p56 proteins were treated with 20μM $NaAsO_2$ for 24 hours and immunostained using anti-HA antibodies (green). Cells were also stained for G3BP1 (red) and with DAPI to detect nuclei (blue). Yellow arrows indicate examples of stress granules. Scale bar = 25μm.

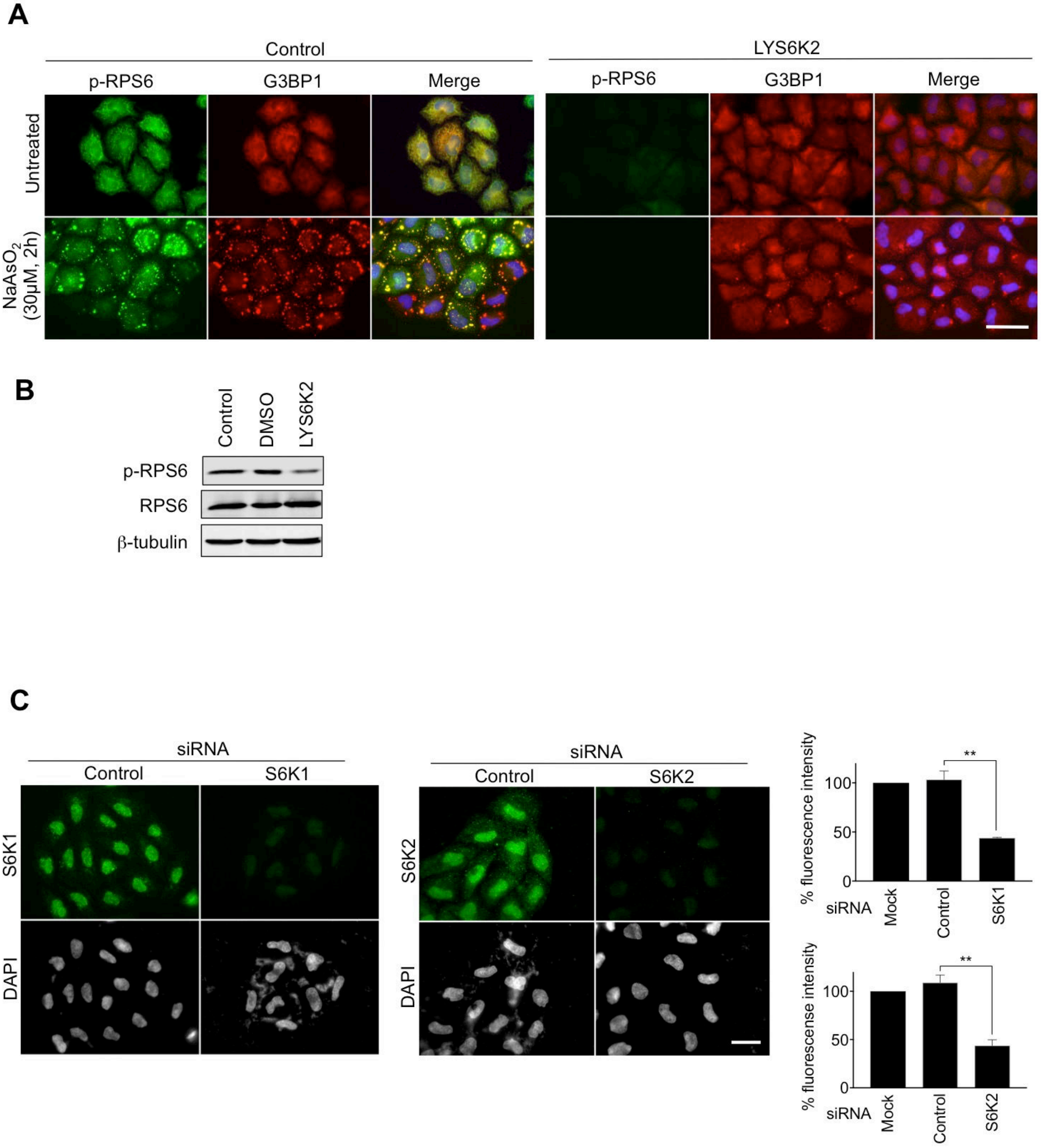

Figure S6: Inhibition of S6 kinase activity reduces stress granule formation. (**A**) Hela cells were pre-treated or not with 0.5µM of LYS6K2 for 12 hours and then treated with or without 30µM $NaAsO_2$ for 2 hours. Cells were immunostained for G3BP1 (red) to assess stress granule formation and p-RPS6 (green) to assess the efficiency of S6 kinase inhibition. Nuclei were stained with DAPI (blue). Scale bar = 50µM. (**B**) Immunoblot of p-RPS6 levels in the indicated conditions. Immunoblots for RPS6 and β-tubulin levels were performed to ensure equal loading of extracts. **(C)** HeLa cells transfected with either S6K1 or S6K siRNAs (S6K1 siRNA-B and S6K2 siRNA-B) were immunostained with anti-S6K antibodies (green). Nuclei were stained with DAPI (grey). Right hand panels are quantifications of S6K1 and S6K2 staining intensity compared to the controls. 100 cells were analysed from each of the 3 biological repeats. Error bars are s.e.m. Data analysed using one-way Anova (**$p<0.0002$). Scale bar = 50µM.

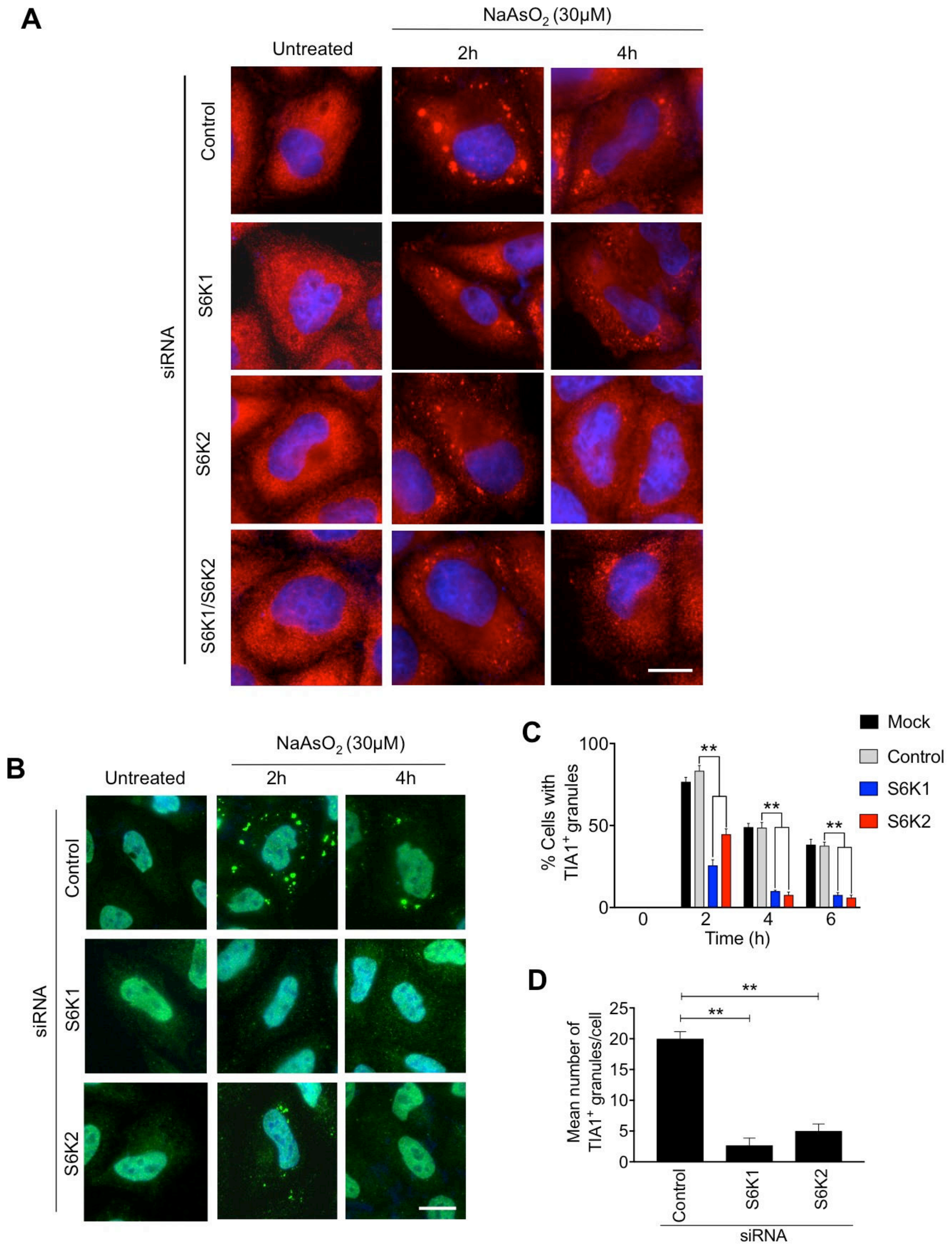

Figure S7: siRNA knock-down of S6K1 and S6K2 reduces stress granule formation. (A, B) Stress granule formation after S6K1 or S6K2 depletion by siRNA and treatment with 30µM $NaAsO_2$ for the indicated times was observed by immunostaining for G3BP1 (red) (A) or TIA1 (green) (B). Nuclei are stained with DAPI (blue). Scale bars = 20µm. (**C**). Quantification of cells with TIA1-positive stress granules after treatment with 30µM $NaAsO_2$ for the indicated times after S6K1 and S6K2 knock-down by siRNA. (**D**) Quantification of the mean number of TIA1-positive granules per cell in those cells where stress granules were detected after 2 hr treatment with 30µM $NaAsO_2$. For all quantifications, 100 cells were analysed in each of 3 biological repeats. Error bars are s.e.m. For panel C, data was analysed using two-way Anova and for panel D, one-way Anova (**p<0.0002).

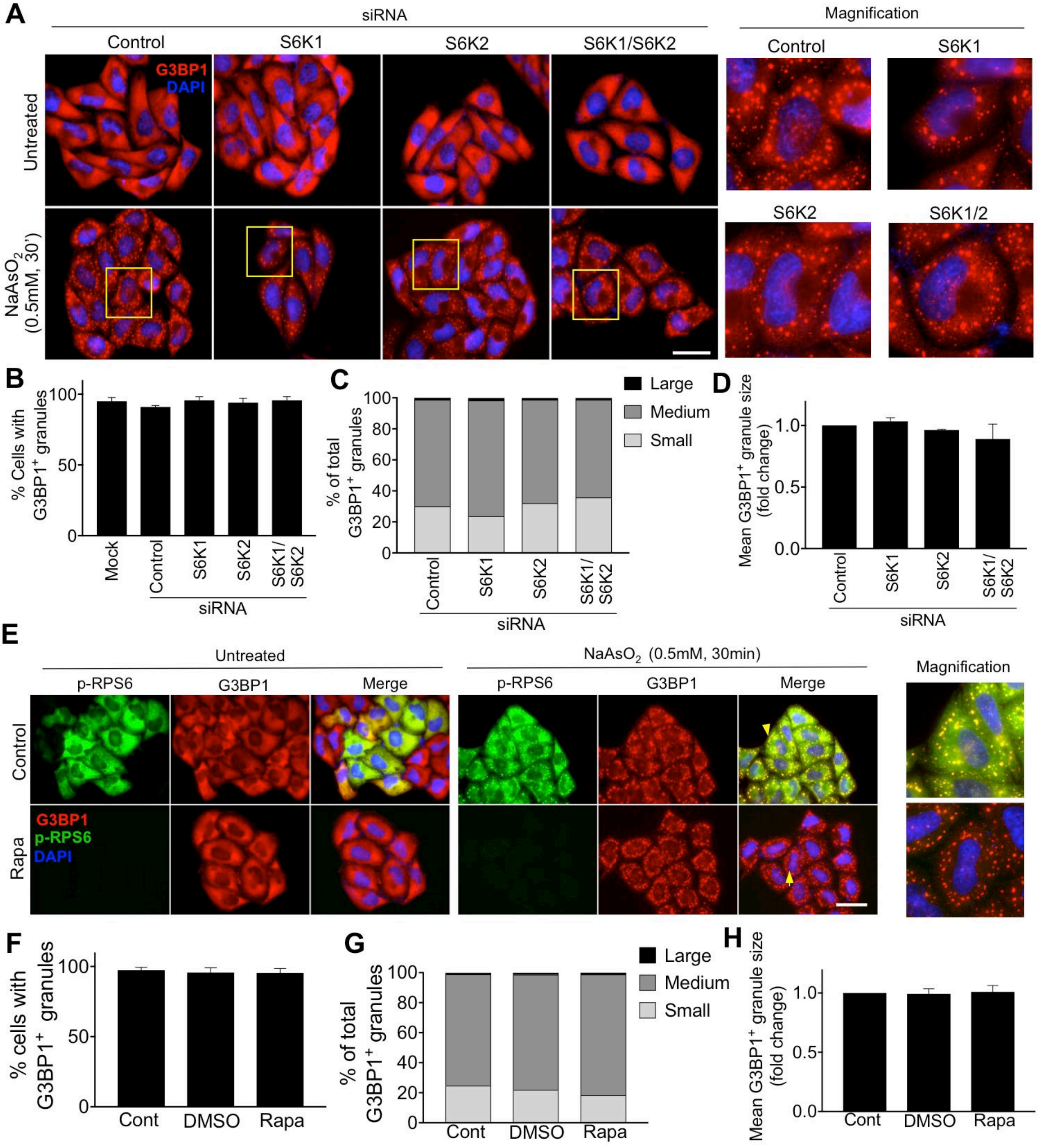

Figure S8: Stress granule formation induced by high arsenite stress is not regulated by S6 kinases. (**A**) HeLa cells transfected with siRNA against S6K1 or S6K2 were treated with 0.5mM $NaAsO_2$ for 30 mins. Stress granules were observed by immunostaining for G3BP1 (red). Nuclei stained with DAPI (blue). Magnifications of the areas boxed in yellow shown in right hand panels. (**B**) Quantification of the number of cells with stress granules after treatment with 0.5mM $NaAsO_2$ for 30 mins and the indicated siRNAs. (**C**) Ratio of small, medium and large stress granules formed in the indicated conditions. (**D**) Mean size of stress granules formed in the indicated conditions. (**E**) HeLa cells were treated with 50nM of Rapamycin (Rapa) for 6 hrs and then with 0.5mM $NaAsO_2$ for 30 mins. Stress granules were observed by immunostaining for G3BP1 (red). Nuclei stained with DAPI (blue). Magnifications of the cell indicated with yellow arrows are shown in right hand panels. (**F**) Quantification of the number of cells with stress granules after treatment with 0.5mM $NaAsO_2$ for 30 mins and the indicated treatments. (**G**) Ratio of small, medium and large stress granules formed in the indicated conditions. (**H**) Mean size of stress granules formed in the indicated conditions. For all experiments, 50 cells were counted in each of 3 biological repeats. Scale bars = 50µm.

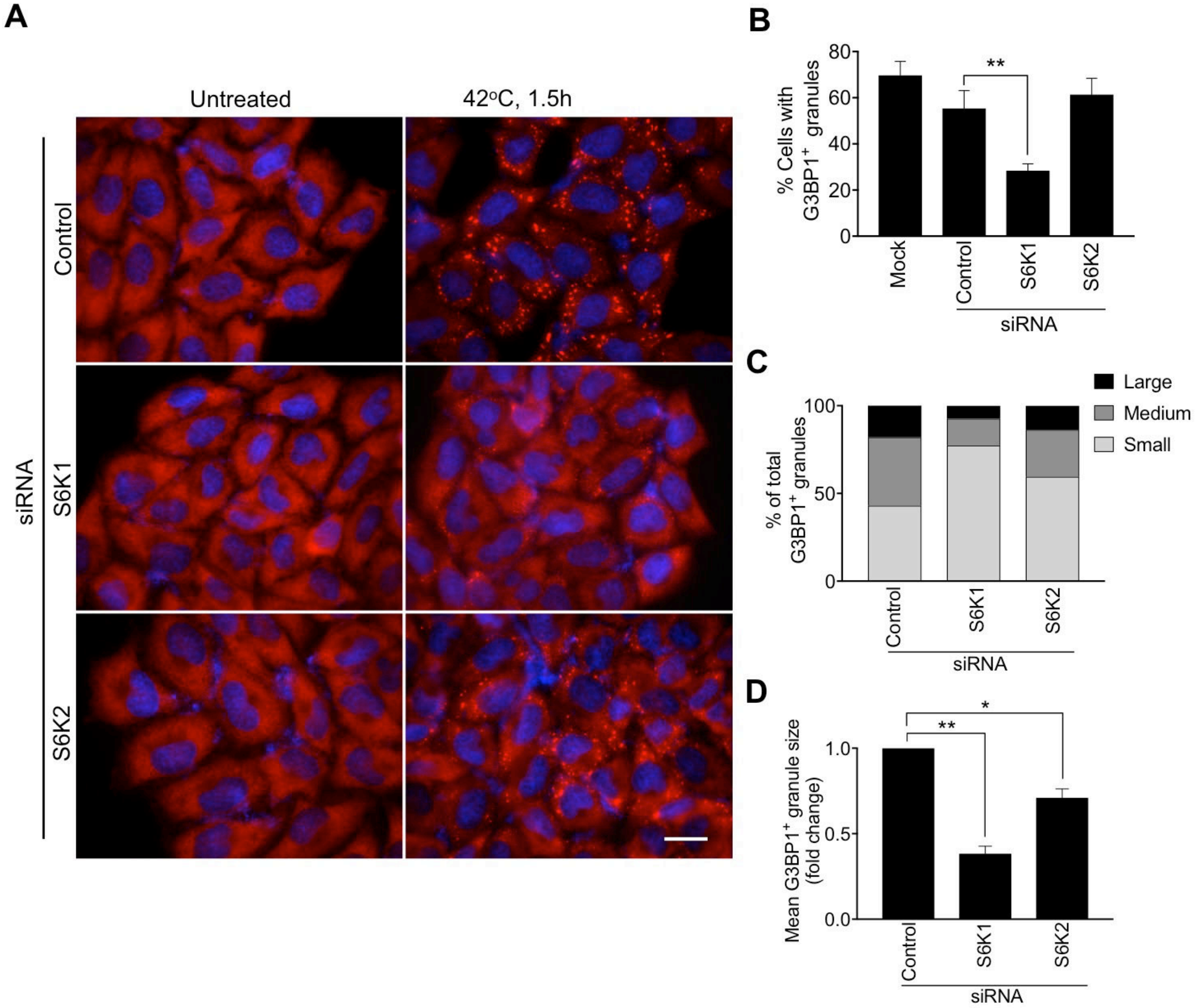

Figure S9: **S6 kinases regulate stress granule formation and size in response to heat.** HeLa cells transfected with siRNAs against S6K1 and S6K2 (S6K1 siRNA-B and S6K2 siRNA-B) were subjected to 42°C for 1.5 hours. (**A**) Stress granules were observed by immunostaining of G3BP1 (red). Nuclei are stained with DAPI (blue). Scale bar = 25µm. (**B**) Quantification of the number of cells with stress granules. (**C, D**) Analysis of the size of stress granules. Ratio of small, medium and large stress granules and the mean size of stress granules formed under the indicated conditions. For all quantifications, 100 cells were analysed in each of 3 biological repeats. For panels B and D, data was analysed using one-way Anova (*p<0.04; **p<0.0002).

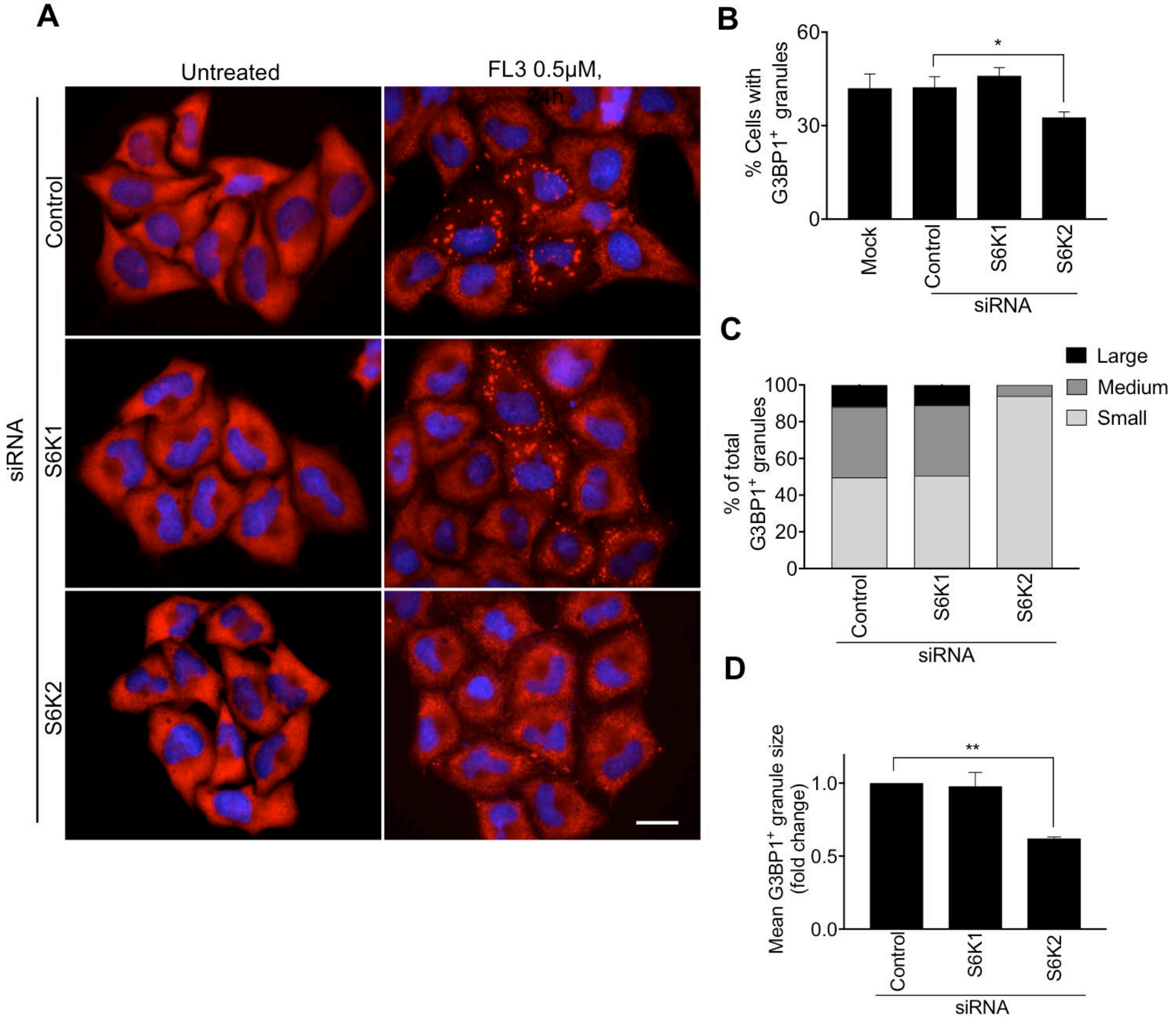

Figure S10: **S6K2 is required for stress granule formation in response to FL3.** (**A**) HeLa cells transfected with siRNAs against S6K1 and S6K2 (S6K1 siRNA-B and S6K2 siRNA-B) were treated with 0.5μM FL3 for 24 hours. Stress granules were observed by immunostaining of G3BP1 (red). Nuclei are stained with DAPI (blue). Scale bar = 25μm. (**B**) Quantification of the number of cells with stress granules. (**C, D**) Analysis of the size of stress granules. Ratio of small, intermediate and large stress granules and the mean size of stress granules formed in the indicated conditions. For all quantifications, 100 cells were analysed in each of 3 biological repeats. For panels B and D, data was analysed using one-way Anova (*p<0.04; **p<0.0002).

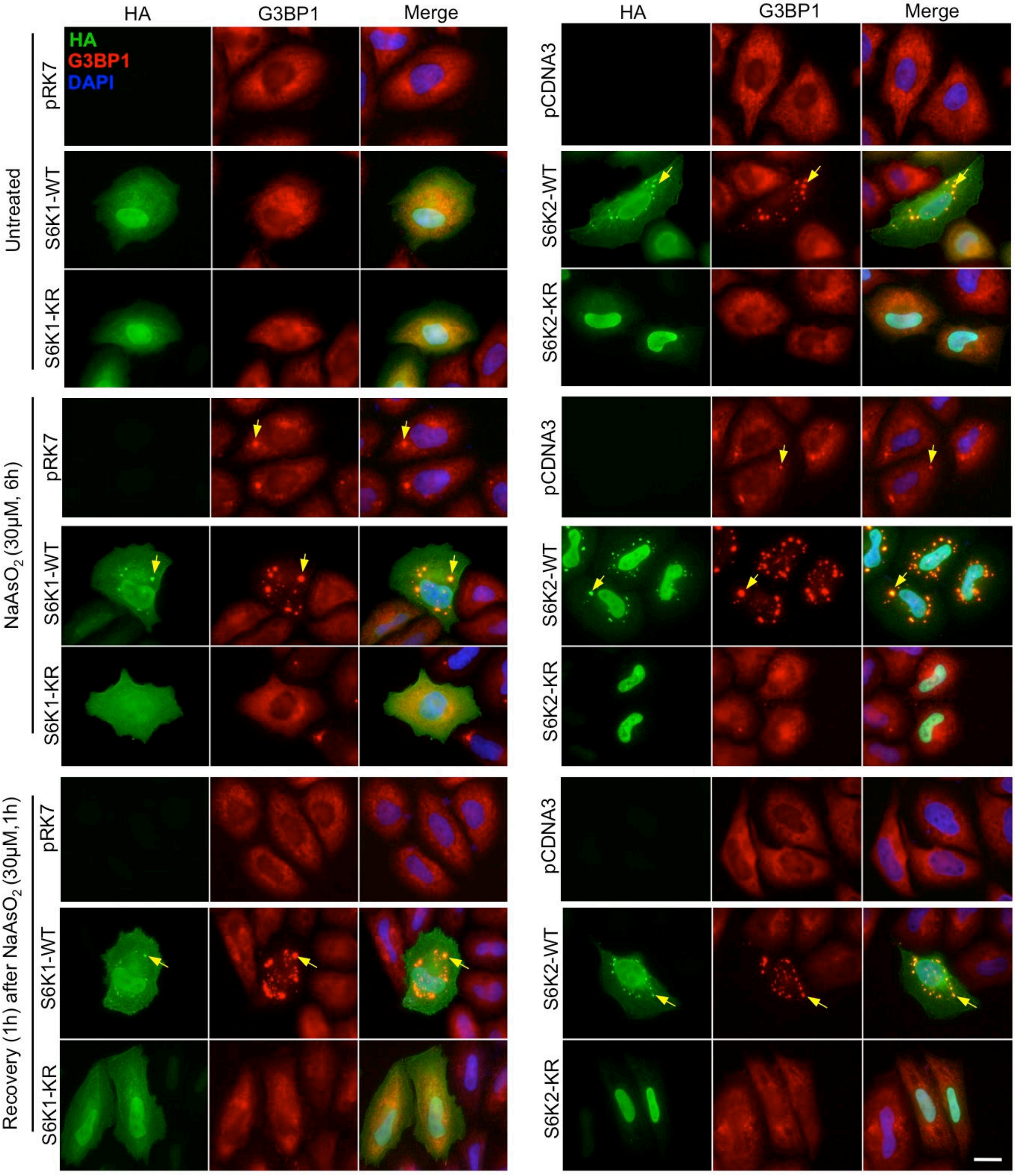

Figure S11: S6K1 and S6K2 promote SG assembly in response to arsenite dependent on their kinase activities. HeLa cells expressing HA-S6K1p70, HA-S6K2p54 or kinase-inactive mutants (KR) were subjected to 30μM $NaAsO_2$ for 6 hours or with 30μM $NaAsO_2$ for 1 hour and left to recover for 1hour. Cells were immunostained for the S6 kinases (HA, green) and G3BP1 (red). Nuclei were stained with DAPI (blue). Yellow arrows indicate examples of stress granules. Scale bar = 25μm.

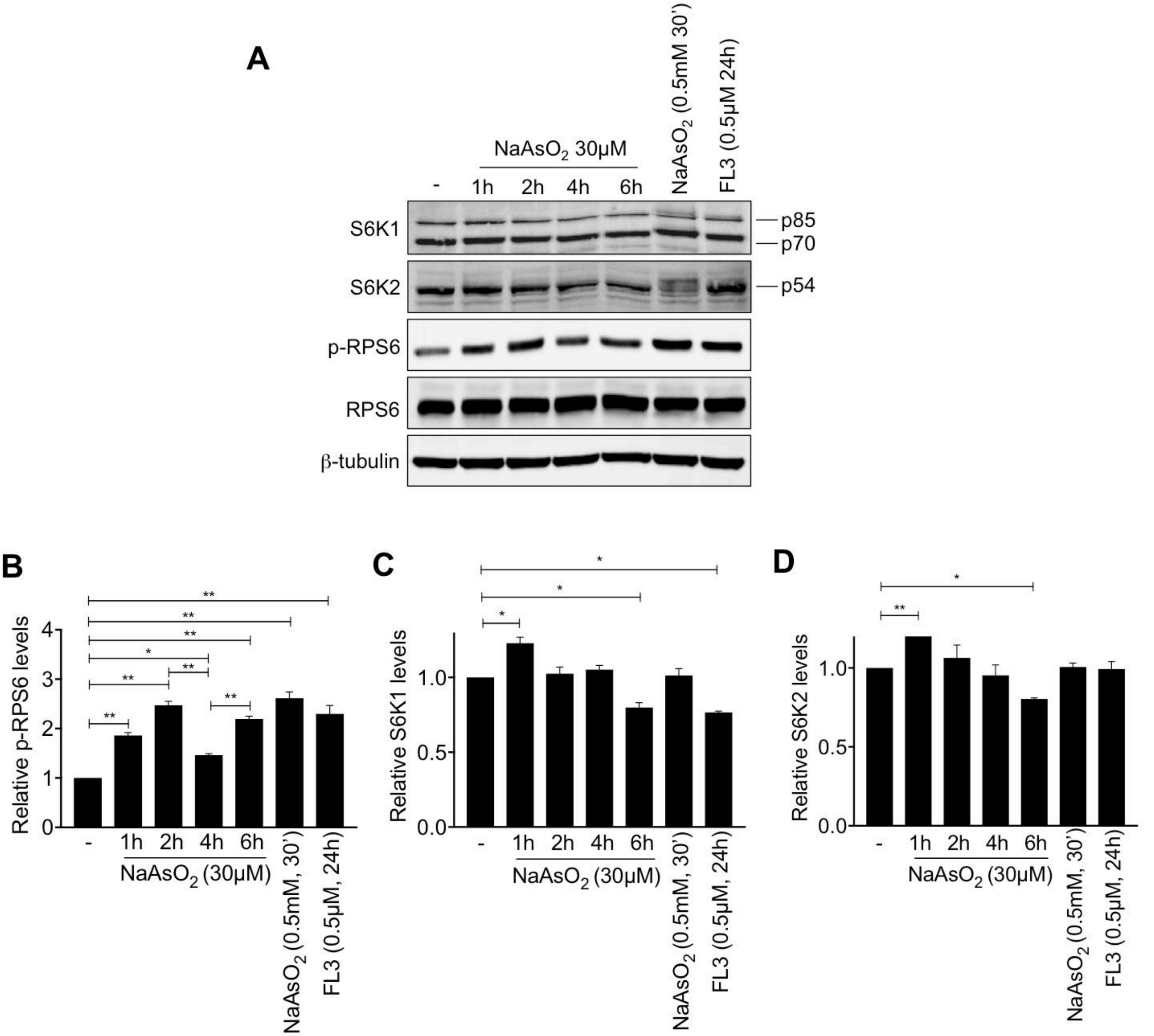

Figure S12: Arsenite and FL3 increase S6K activity (**A**) Protein lysates of HeLa cells treated with 30μM of $NaAsO_2$ for 1, 2, 4 and 6 hrs, 0.5mM $NaAsO_2$ for 30 minutes or FL3 for 0.5μM for 24 hrs were assessed by immunoblotting for phosphorylated RPS6 (p-RPS6), RPS6, S6K1 and S6K2 levels. Quantifications of (**B**) p-RPS6 levels, (**C**) S6K1 levels and (**D**) S6K2 levels are shown. Band intensities were normalised to β-tubulin levels and were quantified from 3 biological repeats. Error bars are s.e.m. Data was analysed using one-way Anova (*p<0.04; **p<0.0002).

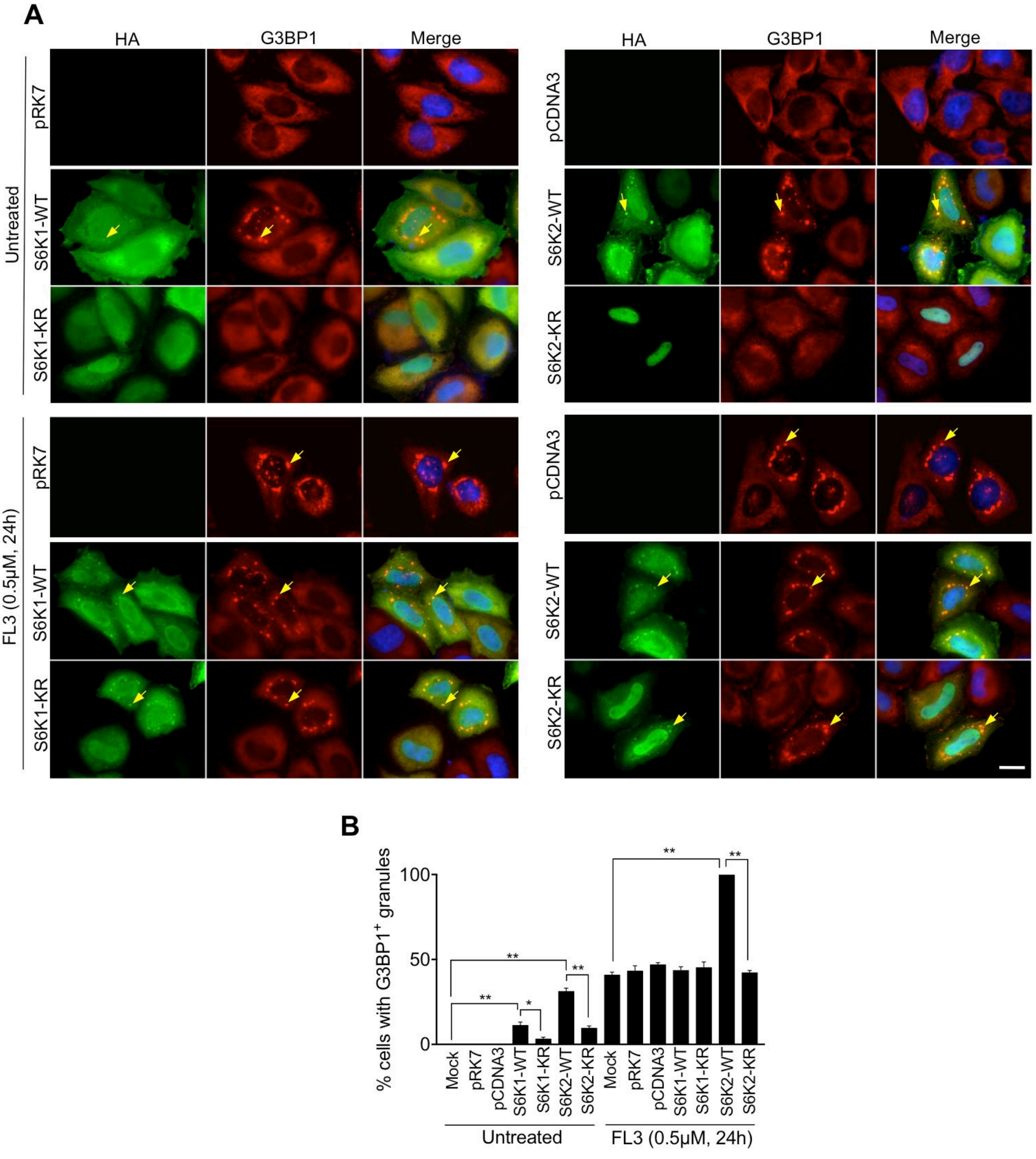

Figure S13: S6K2 promotes stress granule assembly in response to FL3 treatment dependent on its kinase activity. (**A**) HeLa cells expressing HA-S6K1p70, HA-S6K2p54 or kinase-inactive mutants (KR) were subjected to 0.5μM FL3 for 24 hours. Cells were immunostained for the S6 kinases (HA; green) and G3BP1 (red). Nuclei were stained with DAPI (blue). Yellow arrows indicate examples of stress granules. Scale bar = 25μm. (**B**) Quantification of cells with SGs in the indicated conditions. 100 cells were counted per condition in each of three biological repeats. Only cells expressing the HA-tagged S6 kinases were counted. Error bars are s.e.m. Data analysed using one-way Anova (**p*<0.04; ***p*<0.0002).

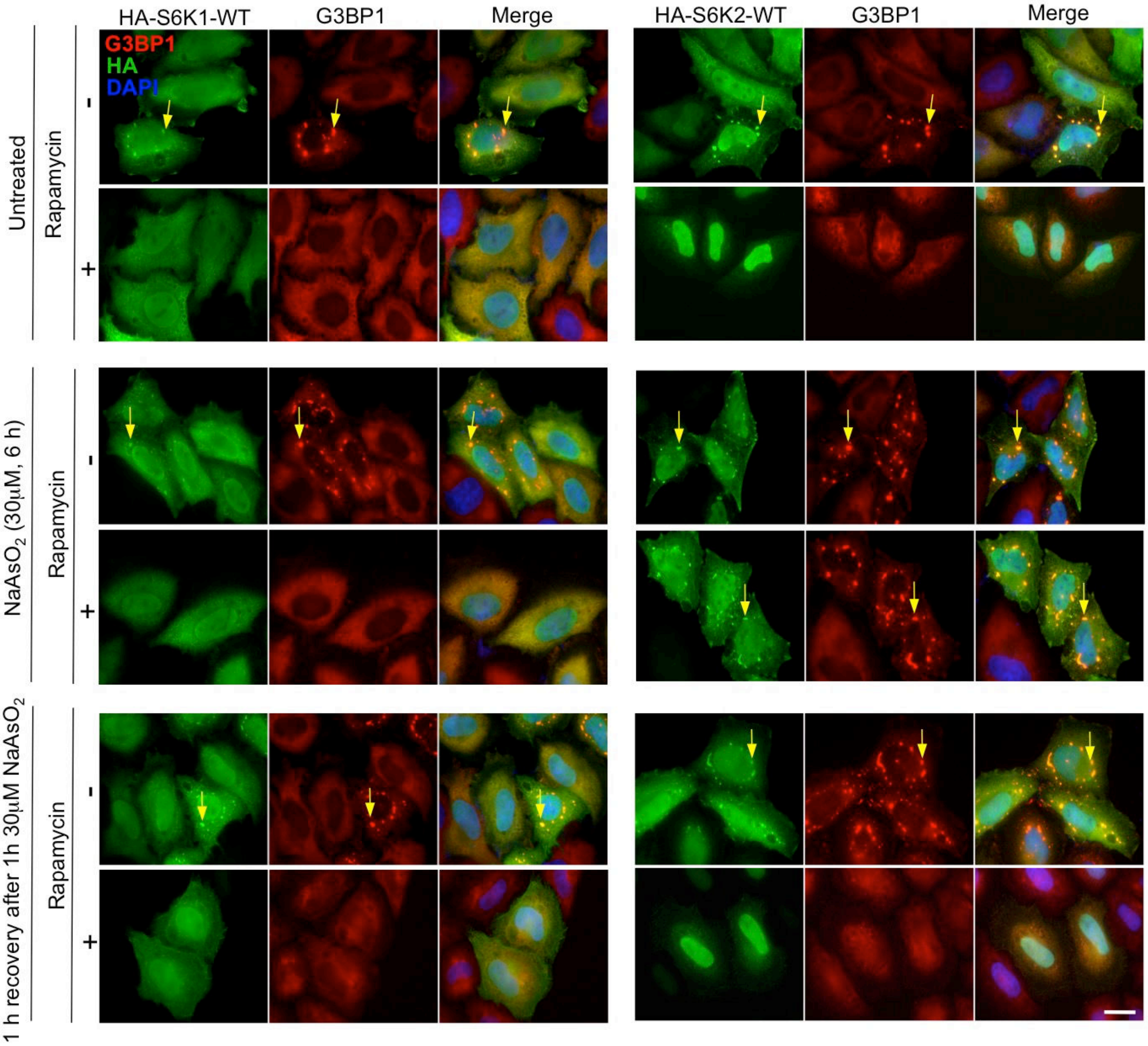

Figure S14: S6 kinases promote SG assembly and persistance dependent on mTORC1 activity. HeLa cells expressing HA-S6K1p70 or HA-S6K2p54 were treated with 50nM Rapamycin for 6h and then were subjected to 30μM $NaAsO_2$ for 6 hours or with 30μM $NaAsO_2$ for 1 hour and left to recover for 1 hour. Cells were immunostained for the S6 kinases (HA, green) and G3BP1 (red). Nuclei were stained with DAPI (blue). Yellow arrows indicate examples of stress granules. Scale bar = 25μm.

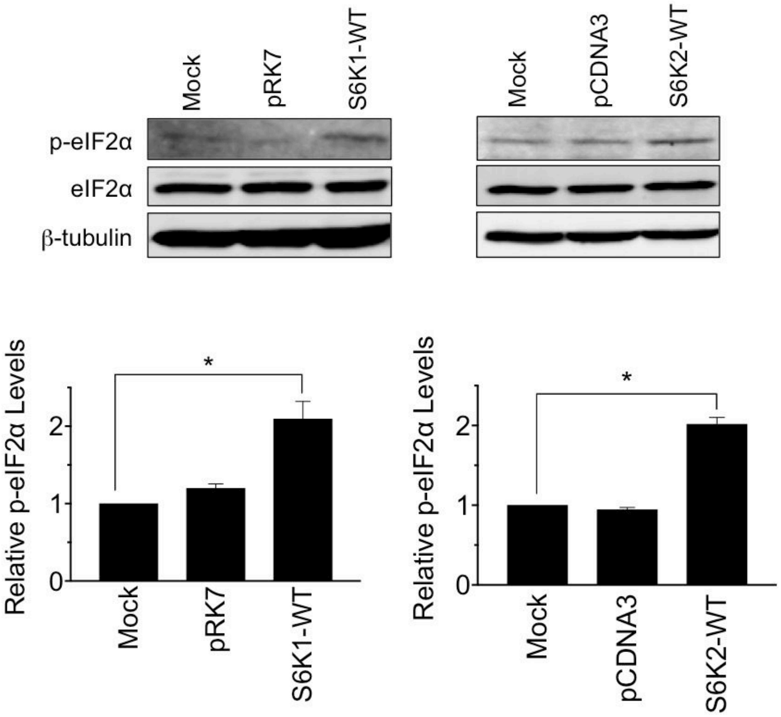

Figure S15: Ectopic expression of S6K1 and S6K2 promotes phosphorylation of eIF2α at Ser 51. Extracts from HeLa cells expressing either HA-S6K1p70 or HA-S6K2p54 were immunoblotted for phosphorylated eIF2α (p-eIF2α), eIF2α and β-tubulin. Images of immunoblots and quantifications from 3 independent experiments are shown. Levels of p-eIF2α were normalised to β-tubulin levels. Error bars are s.e.m. Data was analysed using one-way Anova (*$p<0.04$).

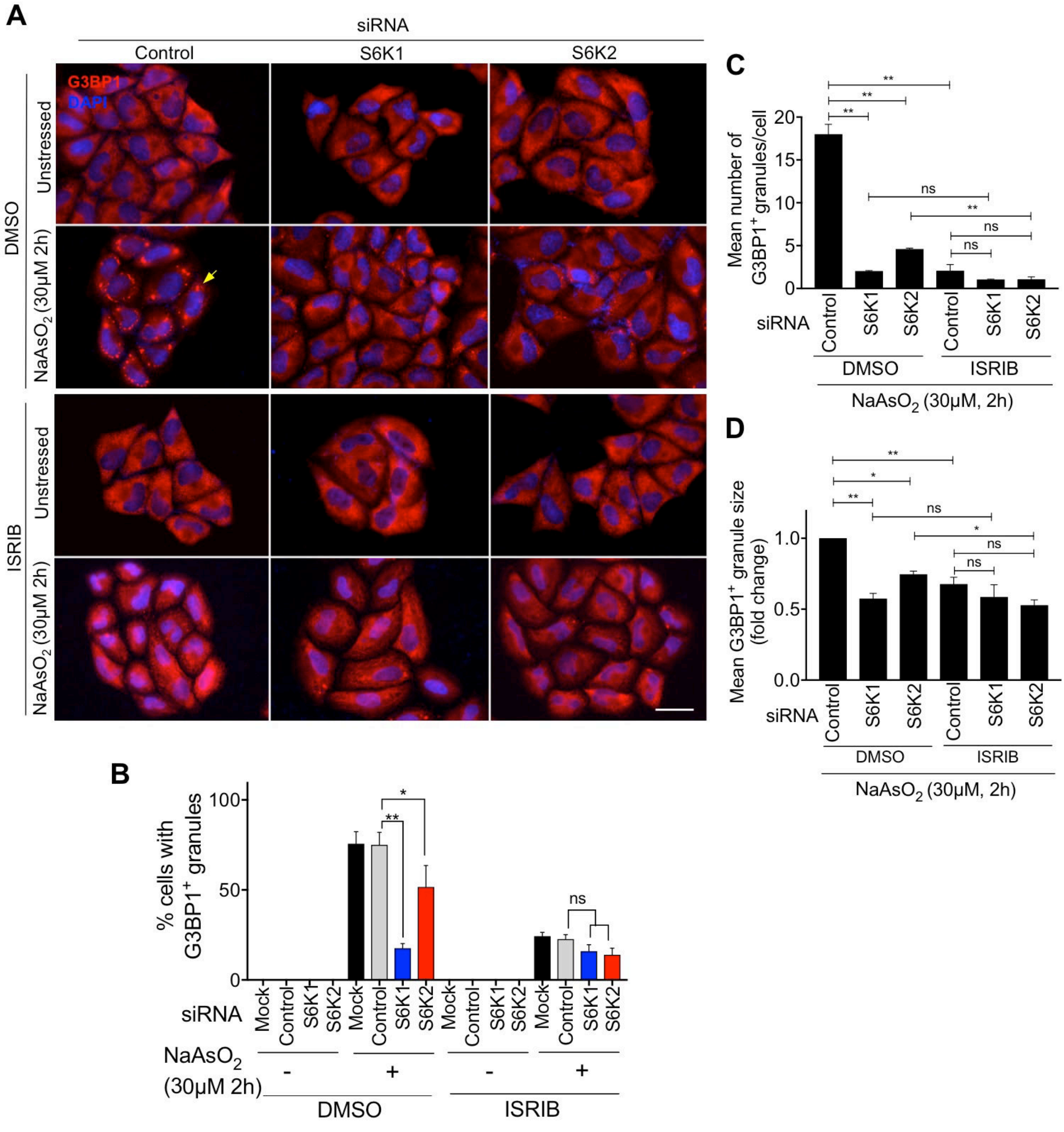

Figure S16: ISRIB does not further suppress stress granule assembly after knock-down of S6K1. (A) HeLa cells transfected with siRNA against S6K1 or S6K2 were treated with or without 30μM $NaAsO_2$ for 2 hours after 2 hours pre-treatment with DMSO or 200nM ISRIB. Cells were immunostained for G3BP1 (red). Nuclei were stained with DAPI (blue). Yellow arrow indicates example of a stress granule. Scale bar = 50μm. (**B**) Quantification of the number of cells displaying stress granules under the indicated conditions. (**C**) Quantification of the mean number of stress granules per cell. (**D**) Quantification of the mean stress granule size under the indicated conditions. For the quantifications, 100 cells were analysed in each of 3 biological repeats. Error bars are s.e.m. Data was analysed using one-way Anova (**p*<0.04; ***p*<0.0002; ns=not significant).

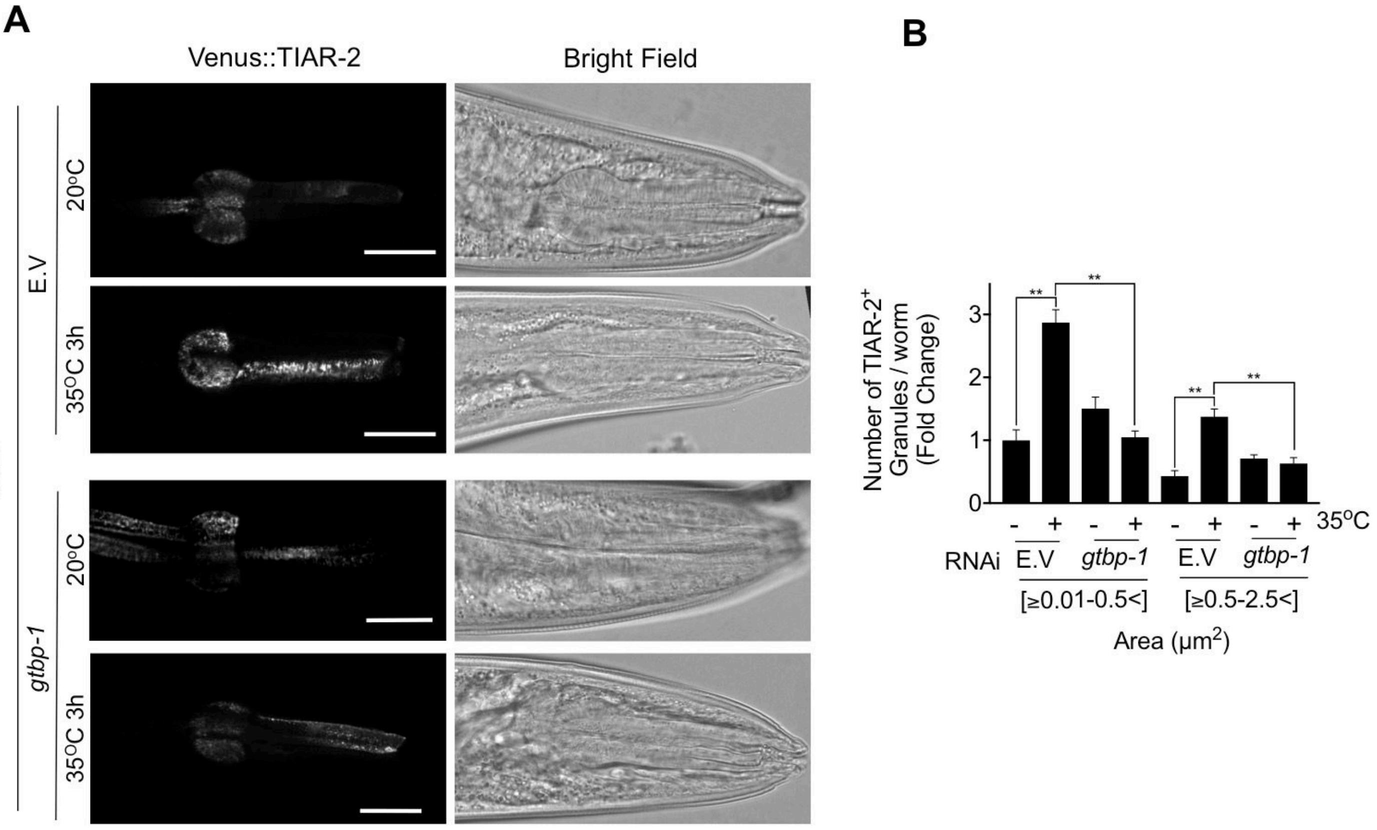

Figure S17: The *C. elegans* stress granule protein GTBP-1 is required for stress granule assembly. (**A**) Worms expressing a pharyngeal Venus::TIAR-2 reporter were fed *gtbp-1* RNAi and subjected to heat shock at 35°C for 3 hours. Images of the pharynx of worms are shown for the indicated conditions. Left hand panels show reporter expression and the right hand panels are corresponding bright field images. Scale bar = 25µm. (**B**) Quantification of the number of TIAR-2 positive granules separated by size, either small (0.01-0.5 μm^2) or large (0.5-2.5 μm^2). 20 worms were analysed per condition in three biological repeats. Data was analysed using one-way Anova (***p*<0.0002)

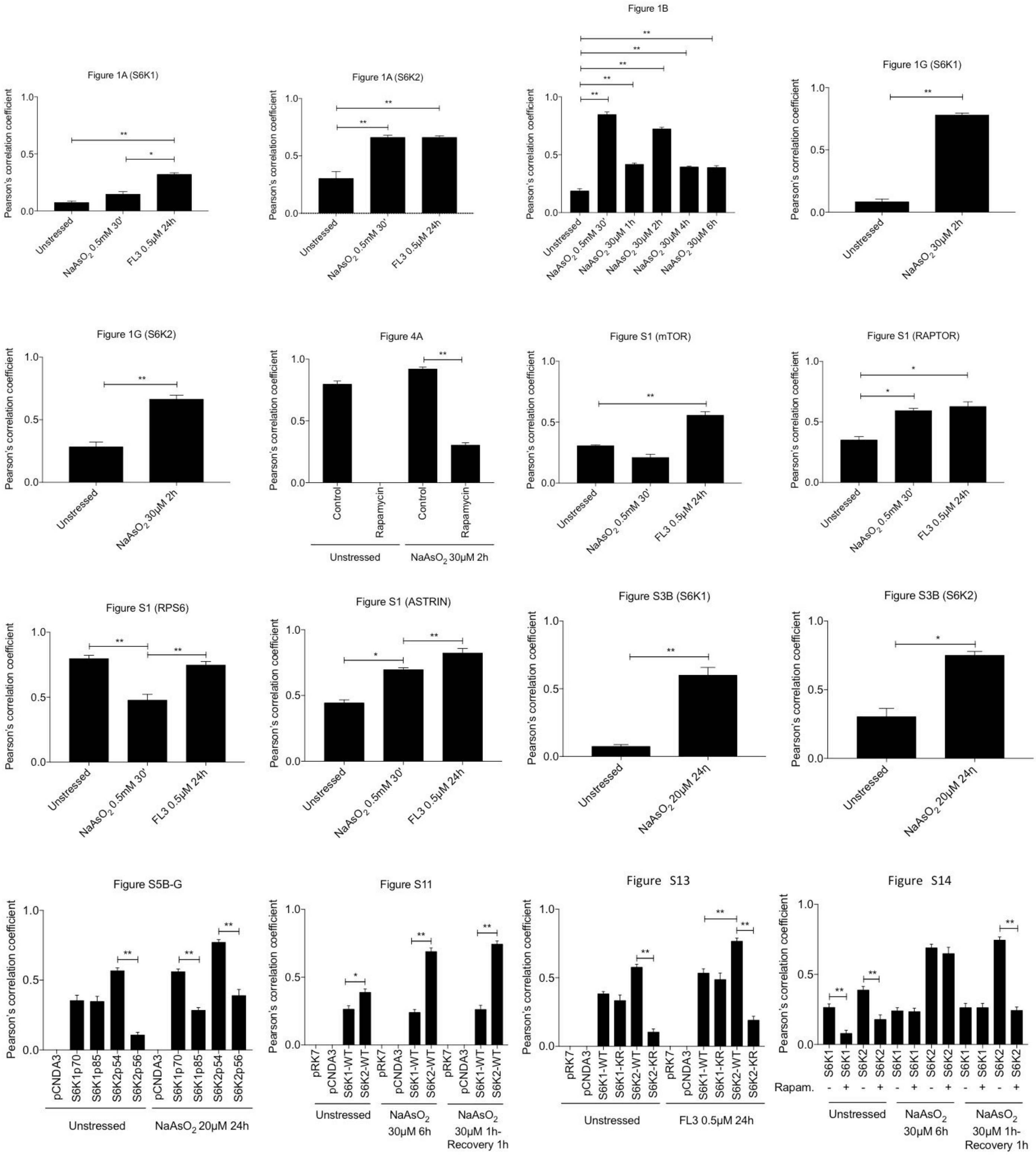

Figure S18: Pearson's correlation coefficient (R) for evaluation of co-localisation with the SG marker protein G3BP1 in immunofluorescence imaging experiments. For statistical analysis 100 cells were analysed in each of 3 biological repeats. Error bars are s.e.m. Data was analysed using one-way Anova (**p*<0.04; ***p*<0.0002; ns=not significant).

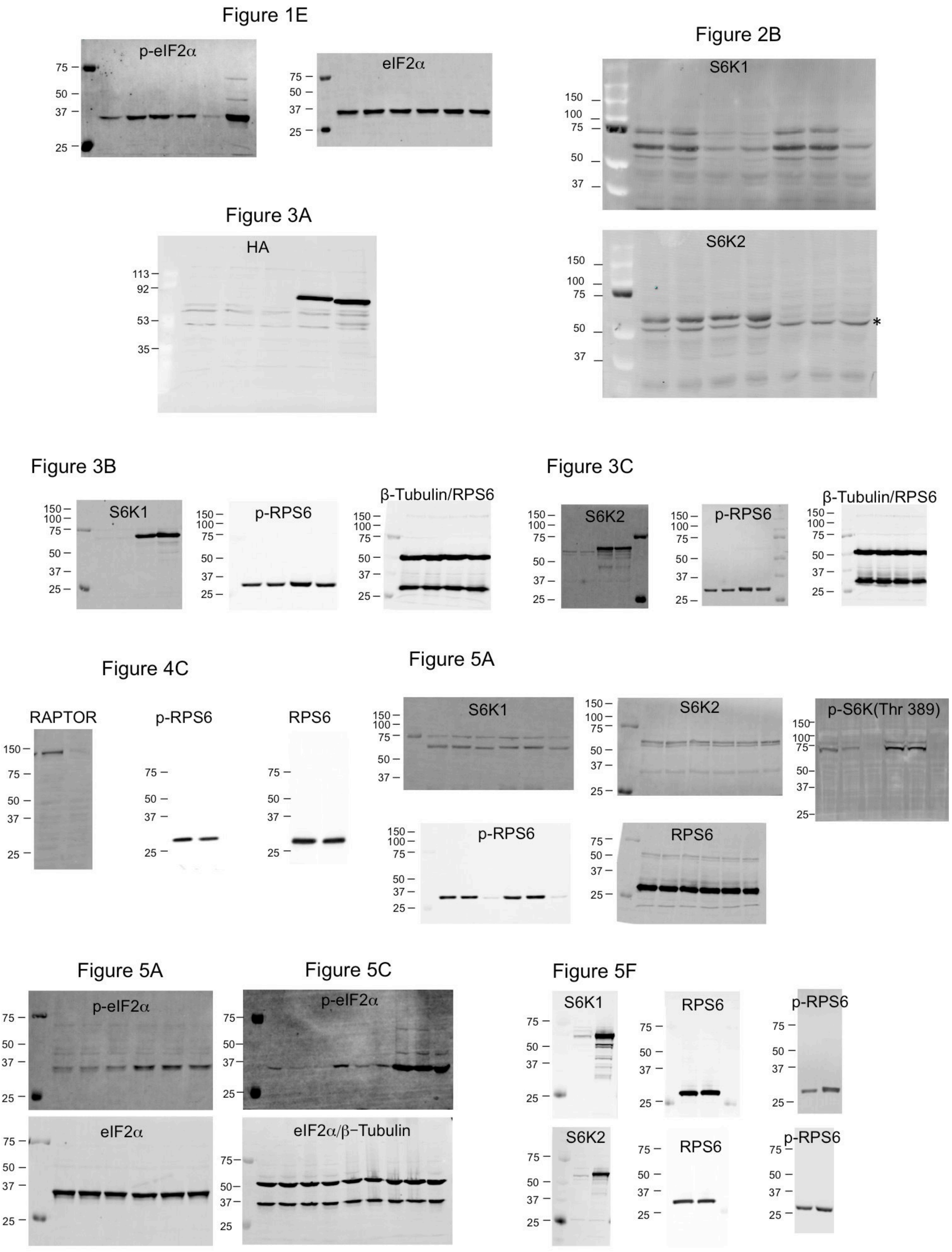

Figure S19: Unprocessed scans of immunoblots in main figurers.
* = non-specific bands. Marker sizes in kDa.

Figure S2B

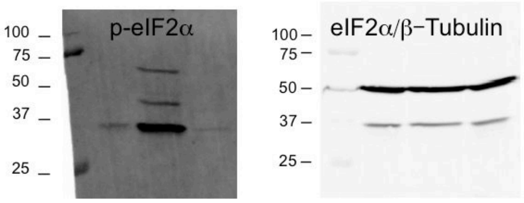

Figure S5B

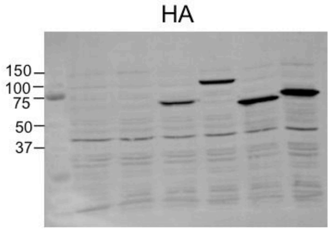

Figure S6B

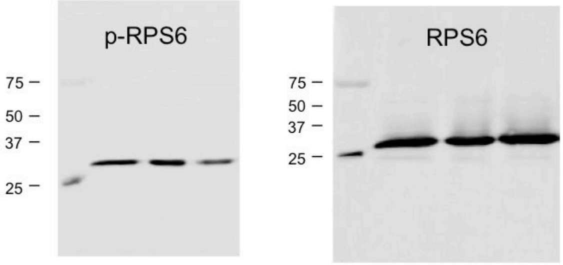

Figure S12

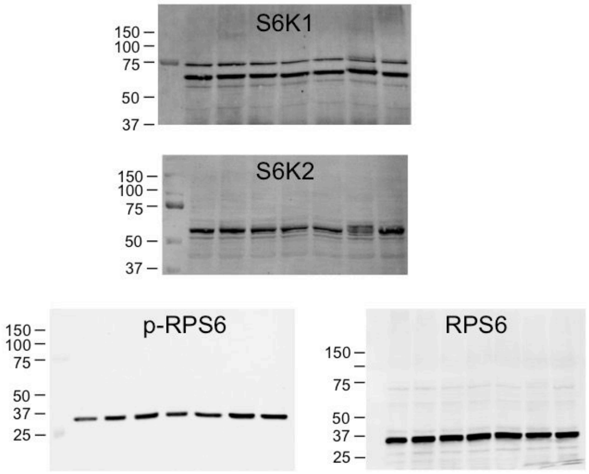

Figure S15

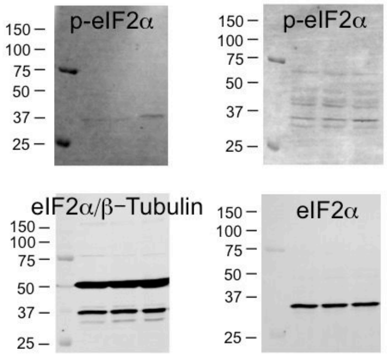

Figure S20: Unprocessed scans of immunoblots in supplementary data.
Marker sizes in KDa.

Antibody	Catalogue Number	Company
Primary		
**eIF2α**	#9722	Cell Signaling
**p-eIF2α (Ser 51)**	#9721	Cell Signaling
**EIF2S1 (phospho-eIF2α Ser51)**	Ab32157	Abcam
**Beta-tubulin**	Ab6046	Abcam
**G3BP1 (H-10)**	SC-365338	Santa Cruz Biotechnology
**TIA1**	Ab2712	Abcam
**RAPTOR**	#2280	Cell Signaling
**S6K1**	#9202	Cell Signaling
**p70 S6 Kinase beta Antibody (S6K2)**	AF2987	R&D Systems
**Phospho-p70 S6 Kinase (Thr389)**	#9205	Cell Signaling
**S6 Ribosomal Protein (5G10) (S6RP)**	#2217	Cell Signaling
**Phospho-S6 Ribosomal Protein (Ser235/236) (2F9)**	#4856	Cell Signaling
**SPAG5 (ASTRIN) (C-18)**	SC-79093	Santa Cruz Biotechnology
**mTOR**	#2972	Cell Signaling
**HA**	H6908	Sigma-Aldrich
**Puromycin, clone 12D10**	#MABE343	EMD Millipore
Secondary Western Blot		
**Goat anti-rabbit IRDye 800CW**	P/N 926-32313	LI-COR
**Goat anti-mouse IRDye 800CW**	P/N 926-32352	LI-COR
Secondary Immunofluorescence		
**Donkey anti-Rabbit IgG (H+L), Alexa Fluor 488**	A-21206	Invitrogen
**Donkey anti-Goat IgG (H+L), Alexa Fluor 488**	A-11055	Invitrogen
**Donkey anti-Goat IgG (H+L), Alexa Fluor 594**	A-11058	Invitrogen
**Donkey anti-Mouse IgG (H+L), Alexa Fluor 594**	A-21203	Invitrogen
**Donkey anti-Mouse IgG (H+L), Alexa Fluor 488**	A-21202	Invitrogen

Table S1: Antibodies used in the study.
